# Pregnancy complications affect kynurenine pathway metabolite concentrations in umbilical cord blood

**DOI:** 10.1186/s12958-025-01436-6

**Published:** 2025-07-21

**Authors:** Michelle Broekhuizen, Heike Allenberg, Claude P. van der Ley, Sofie K. M. van Zundert, Zongye Cai, Martijn van Faassen, Daphne Merkus, A. H. Jan Danser, Anja Lange, Irwin K. M. Reiss, Matthias Heckmann

**Affiliations:** 1 Division of Neonatology, Department of Pediatric and Neonatal Intensive Care, Erasmus MC University Medical Center, Rotterdam, The Netherlands; 2 Division of Pharmacology and Vascular Medicine, Department of Internal Medicine, Erasmus MC University Medical Center, Rotterdam, The Netherlands; 3 Department of Neonatology and Pediatric Intensive Care, University Medicine Greifswald, Greifswald, Germany; 4 Department of Laboratory Medicine, University Medical Center Groningen, University of Groningen, Groningen, The Netherlands; 5 Department of Obstetrics and Gynecology, Erasmus MC University Medical Center, Rotterdam, The Netherlands; 6 Department of Clinical Chemistry, Erasmus MC University Medical Center, Rotterdam, The Netherlands; 7 Department of Cardiology, Erasmus MC University Medical Center, Rotterdam, The Netherlands; 8 Walter Brendel Center of Experimental Medicine, University Clinic Munich, Ludwig Maximillian University (LMU) Munich, Munich, Germany; 9 Interfaculty Center for Endocrine and Cardiovascular Disease Network Modelling and Clinical Transfer (ICONLMU), Ludwig Maximillian University (LMU) Munich, Munich, Germany; 10 German Center for Cardiovascular Research (DZHK), Munich Heart Alliance (MHA), Partner Site Munich, Munich, Germany; 11 German Centre for Child and Adolescent Health (DZKJ), Partner site Greifswald/Rostock, Greifswald, Germany

**Keywords:** Kynurenine pathway, Tryptophan, Pregnancy, Pregnancy complication, Umbilical cord blood, Fetus

## Abstract

**Background:**

Tryptophan and its kynurenine pathway (KP) metabolites play key roles in modulating the immune system and vasculature, and exhibit both pro- and antioxidant properties, making them crucial for a healthy pregnancy and fetal development. Disruptions in the KP may impact both prenatal and postnatal health, however, data on fetal KP metabolite concentrations and their alterations in pregnancy-related disorders remain scarce. This study aims to investigate the association between pregnancy complications and KP metabolite concentrations in umbilical cord blood.

**Methods:**

Pregnancies complicated by preeclampsia (*n* = 40), fetal growth restriction (FGR, *n* = 33), pregestational diabetes mellitus (DM, *n* = 42), gestational diabetes mellitus (GDM, *n* = 61), and amniotic infection syndrome (AIS, *n* = 47) were included, along with 410 controls matched in a 1:2 ratio using Mahalanobis nearest-neighbor matching from a prospective birth cohort study. Tryptophan, kynurenine, anthranilic acid, 3-hydroxykynurenine, 3-hydroxyanthranilic acid, kynurenic acid, xanthurenic acid, quinolinic acid, picolinic acid, and nicotinic acid were measured in umbilical cord blood using liquid chromatography-tandem mass spectrometry (LC-MS/MS). Differences in metabolite concentrations were analyzed using unpaired t-tests and linear regression models to control for potential confounders.

**Results:**

Tryptophan concentrations were decreased in cases of preeclampsia and DM. We identified elevated levels of 3-hydroxykynurenine in preeclampsia, kynurenine in GDM, and nicotinic acid in both FGR and DM. Quinolinic acid levels were also higher in preeclampsia and GDM, although this was not significant after adjusting for confounding variables. We observed no changes in KP metabolites in AIS.

**Conclusion:**

This study identified distinct alterations in umbilical cord blood KP metabolite concentrations in pregnancies with preeclampsia, FGR, DM, and GDM, but not AIS. This suggests differential regulation and activation of the KP depending on the pregnancy disorder. Such changes may influence maternal and infant health and could play a role in fetal programming, with potential long-term effects on child development and health.

**Supplementary Information:**

The online version contains supplementary material available at 10.1186/s12958-025-01436-6.

## Background

**Fig. 1:**
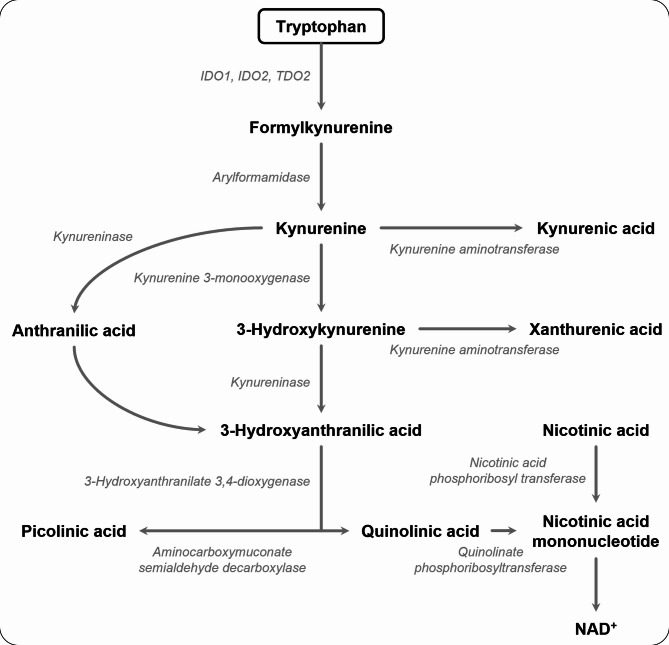
The kynurenine pathway metabolites (in bold) and enzymes (in italic). IDO indicates indoleamine 2,3-dioxygenase; TDO, tryptophan 2,3-dioxygense; Adapted from Van Zundert, Broekhuizen et al. [5]

Fetal development depends on an efficient placental exchange of nutrients, oxygen, and waste products. Tryptophan (L-tryptophan) is an essential amino acid that is required for protein synthesis and thus indispensable for fetal and placental growth. However, more than 95% of tryptophan is metabolized via the kynurenine pathway (KP), yielding kynurenine and over 10 downstream metabolites (Fig. 1). These metabolites have diverse roles in processes that are critical during pregnancy, including immune modulation, pro- and antioxidant effects, and regulation of vascular development and tone [1]. Adequate regulation of the placental KP is essential for a healthy pregnancy. Inhibition of this pathway in pregnant mice leads to fetal loss [2–4]. In humans, KP disturbances in maternal blood and placental tissue have also been linked to various pregnancy complications [5].

Placental KP metabolism may also influence the transfer of tryptophan and KP metabolites from mother to fetus, subsequently affecting fetal development. The human placenta is one of the few organs with constitutive expression of the first KP enzyme indoleamine 2,3-dioxygenase (IDO), along with other downstream KP enzymes [1, 6, 7]. Our previous work confirmed that the human placenta transfers tryptophan from the maternal to the fetal circulation, while simultaneously metabolizing a portion into KP metabolites which are also released into the fetal circulation [8]. The conversion of tryptophan into kynurenine also has functional implications in the placenta. We and others have shown that tryptophan induces vasodilation in fetoplacental arteries in an IDO1-dependent manner, which was altered in the placenta-related pregnancy complication preeclampsia [8, 9]. However, the extent to which maternal and placental KP dysregulation influences fetal circulating concentrations of these metabolites remains unclear. This knowledge gap is critical, given the essential role of tryptophan and KP metabolites in fetal development and long-term health.

Through a systematic review of literature, we identified that a tightly regulated balance of KP metabolites is essential for physiological pregnancy. Disruptions in either direction may be associated with adverse maternal pregnancy and fetal outcomes [5]. However, data on KP metabolite concentrations in the fetal circulation and how these are affected by pregnancy complications remain limited. We hypothesized that alterations in maternal and placental KP metabolism due to pregnancy complications, could affect fetal exposure to tryptophan and its downstream KP metabolites. Such disturbances may impact infant health both before and after birth. Therefore, in this study we aimed to investigate the association between pregnancy-related complications, including preeclampsia, fetal growth restriction (FGR), diabetes, and amniotic infection syndrome (AIS), and KP metabolite concentrations in umbilical cord blood.

## Methods

### Study cohort

This study was conducted using data from the prospective population-based birth cohort study Survey of Neonates in Pomerania (SNiP) located in Western-Pomerania, Germany. A comprehensive description of the cohort has previously been published [10–12]. SNiP consists of two independent cohorts. This data analysis made use of plasma samples that were collected during the second cohort SNiP-II, which recruited participants at the time of birth between the years 2013 and 2017 (clinical trial number: not applicable) [12]. Samples were stored at -80 °C until analysis.

### Patient selection

We performed case-control matching on the total study cohort (*n* = 1145) based on fetal sex, gestational age, and maternal body mass index (BMI). Patients with missing BMI, or gestational age were excluded (*n* = 21). The remaining samples were split into two groups: (1) cases (*n* = 212) presenting with one or multiple of the following conditions: preeclampsia (*n* = 42), FGR, *n* = 35, diabetes mellitus before and during pregnancy (DM, *n* = 45), gestational diabetes mellitus (GDM, *n* = 62), AIS (*n* = 50), and (2) controls (*n* = 912) presenting without any of the before mentioned conditions. Every case was matched to two controls based on potential cofounders that we identified in literature: fetal sex (exact) [13], gestational age [5, 14], and BMI [15, 16] using mahalanobis nearest-neighbour matching. The quality of the matching procedure was assessed by studying the spread in sex, gestational age and BMI between the cases and controls, also within the separate study cohorts: preeclampsia, FGR, diabetes, and AIS. During sample selection preceding the liquid chromatography tandem mass spectrometry (LC-MS/MS) analysis, 11 samples were missing from the case group, leading to the exclusion of these cases and their respective controls from further analysis, resulting in the final cohort of 410 controls and 205 cases including: preeclampsia (*n* = 40), FGR (*n* = 33), DM (*n* = 42), GDM (*n* = 61), AIS (*n* = 47).

The obstetrician in charge diagnosed preeclampsia, FGR, DM, GDM and AIS according to in-house and German national guidelines and coded according to International Classification of Diseases (ICD)-10. Preeclampsia was diagnosed based on the German national guideline criteria, that is, new-onset hypertension (systolic blood pressure ≥ 140 mm Hg or diastolic blood pressure ≥ 90 mm Hg) and proteinuria (≥ 300 mg/24 h detected in 24-h urine or > 30 mg/mmol protein-creatinine ratio in a random urine sample occurring after the 20th completed week of pregnancy [17]. Pregestational DM (type 1 or type 2) was documented according to the medical history of the mother. GDM was diagnosed based on the maternity directive of the Federal Joint Committee [18]. FGR was defined as a birth weight below the 10th percentile based on growth curves by Voigt et al. [19]. AIS was diagnosed based on the presence of clinical symptoms including maternal fever, unrestrained contractions, uterine tenderness, and elevated laboratory markers of inflammation. Chart reviews were conducted to collect all required information from medical records, including the prenatal care booklet (maternity records), and hospital files.

### KP metabolites measurements

Tryptophan and other KP metabolites were analyzed using isotope dilution liquid chromatography in combination with tandem mass spectrometry (LC-MS/MS) using an adapted method as previously described [20, 21]. Fifty microliter of sample was used. An ACQUITY 2D-ultra-performance LC system (Waters) in combination with XEVO-TQ-XS mass spectrometer system in positive ionization mode was used. The chromatography was performed on an ACQUITY UPLC HSS PFP 1.8 μm 2.1 × 100 mm column (Waters). The method was validated according to guidelines from the Netherlands Society for Clinical Chemistry and Laboratory Medicine and the ISO 15189:2012 standard [22]. Each analyte had its own stable isotope labelled standard, [^13^C_11_]-tryptophan, [^13^C_6_]-kynurenine, [^13^C_6_]-anthranilic acid, [^13^C_6_]-3-hydroxykynurenine, [^13^C_6_]-3-hdroxyanthranilic acid, [^2^H_5_]-kynurenic acid, [^2^H_4_]-xanthurenic acid, [^2^H_3_]-quinolinic acid, [^2^H_4_]-picolinic acid, and [^13^C_6_]-nicotinic acid (purchased from Cambridge Isotope Laboratories, Alsachim, Merck, and CDN Isotopes).

The samples from this study were analyzed in eight different batches. The same quality control samples (covering three distinct concentration levels for each analyte) were analyzed with each batch. Interassay imprecisions for these eight batches were as follow: tryptophan < 4.3%, kynurenine < 4.2%, anthranilic acid < 4.9%, 3-hydroxykynurenine < 7.5%, 3-hydroxyanthranilic acid < 8.7%, kynurenic acid < 4.5%, xanthurenic acid < 4.7%, quinolinic acid < 4.6%, picolinic acid < 4.8%, nicotinic acid < 5.3%. Lower limits of quantification for tryptophan and the KP metabolites were as follows: tryptophan 0.5 µmol/l, kynurenine 0.05 µmol/l, anthranilic acid 1.4 nmol/l, 3-hydroxykynurenine 1.6 nmol/l, 3-hydroxyanthranilic acid 2.9 nmol/l, kynurenic acid 1.7 nmol/l, xanthurenic acid 0.5 nmol/l, quinolinic acid 14 nmol/l, picolinic acid 1.3 nmol/l, nicotinic acid 0.03 nmol/l. The concentration of nicotinic acid was below the limit of quantification in 57 of the control samples and 22 of the case samples. These samples were left out of the nicotinic acid analysis, under the assumption that the differences between groups would not be significantly affected as the proportion of missing samples was similar between the controls and cases (13.9% and 10.7% respectively).

### Statistical analyses

All statistical analyses were performed in R version 4.2.1 using the packages matchit, ggplot, ggcorrplot, rstatix, and evolqg [23–28].

The concentrations of KP metabolites were natural log transformed to obtain an approximate normal distribution. The complete cohort was used to study correlations between the different KP metabolite concentrations in a correlation matrix. Nicotinic acid was excluded from this analysis due to the missing values. The effect of the pregnancy complications on these correlations were studied by repeating this analysis separately for the disease groups: preeclampsia, FGR, DM, GDM, and AIS. The correlation analyses were performed on the data without extreme univariate outliers, defined as any value above 3rd quartile + 3 * interquartile range or below 1st quartile – 3 * interquartile range, since these outliers were found to have a significant impact on the correlations.

The concentrations were compared between the groups and their matched controls using unpaired t-tests on the natural log transformed data, corrected for multiple testing using the Benjamini-Hoghberg procedure. To study the potential effects of confounders two linear models were constructed: Model 1 was only adjusted for the grouping variable, Model 2 was additionally adjusted for potential confounders based on literature: fetal sex, BMI, gestational age and smoking status [13–16, 29]. Since extreme univariate outliers were identified to have a significant impact on the models, the presented models are those based on the data from which extreme univariate outliers were removed.

Data were presented as median (1st quartile– 3rd quartile), number (percentage), or as estimate [95% confidence interval]. Results were considered statistically significant different if *P* < 0.05.

## Results

### Distribution of KP metabolites

The clinical descriptives of the full cohort can be found in Table 1. The median age of the total study population was 30.0 (27.0, 33.0) years, with a normal median BMI of 24.8 (21.8, 28.6) kg/m^2^. The women delivered at a median gestational age of 39.0 (38.0, 40.0) weeks. 10.1% of the women smoked during the last four weeks of pregnancy. Case-control matching was successful as BMI, gestational age and fetal sex did not differ between the groups. Compared with controls, the case group displayed a higher incidence of caesarean sections (44.3% versus 25.1%) as well as a lower median birth weight (3270.0 versus 3400.0 g).

**Table 1:** Clinical descriptives of the full study cohort

Variable	Controls (*n* = 410)	Cases (*n* = 205)	All (*n* = 615)	*P*-value
**Age mom**	30.0 (27.0, 33.0)	30.0 (27.0, 34.0)	30.0 (27.0, 33.0)	0.362
**BMI (kg/mm** ^**2**^ **)**	24.6 (21.8, 28.1)	25.0 (21.8, 30.1)	24.8 (21.8, 28.6)	0.242
**Smoking**				0.382
never	164 (40.9%)	76 (38.0%)	240 (39.9%)	
former	201 (50.1%)	99 (49.5%)	300 (49.9%)	
last 4 weeks of pregnancy	36 (9.0%)	25 (12.5%)	61 (10.1%)	
**Cesarean section**	102 (25.1%)	89 (44.3%)	191 (31.4%)	**< 0.001**
**Gestational age (weeks)**	39.0 (38.0, 40.0)	39.0 (38.0, 40.0)	39.0 (38.0, 40.0)	0.314
**Sex girl**	186 (45.4%)	93 (45.4%)	279 (45.4%)	1
**Birth weight (grams)**	3400.0 (3013.8, 3750.0)	3270.0 (2750.0, 3720.0)	3360.0 (2955.0, 3740.0)	**0.017**
**Placenta weight (grams)**	570.0 (492.5, 660.0)	560.0 (470.0, 650.0)	560.0 (490.0, 650.0)	0.14
**NICU admission**	67 (16.5%)	64 (31.5%)	131 (21.5%)	**< 0.001**
**Preeclampsia**	0 (0.0%)	40 (19.5%)	40 (6.5%)	
**FGR**	0 (0.0%)	33 (16.1%)	33 (5.4%)	
**GDM**	0 (0.0%)	61 (29.8%)	61 (9.7%)	
**DM**	0 (0.0%)	42 (20.5%)	42 (6.6%)	
**AIS**	0 (0.0%)	47 (22.9%)	47 (7.4%)	

Abbreviations: BMI, body mass index; NICU, neonatal intensive care unit; FGR, fetal growth restriction; GDM, gestational diabetes mellitus; DM, pregestational diabetes mellitus; AIS, amniotic infection syndrome

Maternal and pregnancy characteristics had a significant effect on the concentrations of several KP metabolites as illustrated by Supplementary Figures S1–S6. Pregnancy duration, BMI and fetal sex substantially affected the umbilical cord blood concentrations of all KP metabolites apart from kynurenine and nicotinic acid. A higher gestational age positively associated with tryptophan, kynurenic acid and xanthurenic acid concentrations, but negatively with anthranilic acid, 3-hydroxykynurenine, 3-hydroxyanthranilic acid, quinolinic acid and picolinic acid concentrations. An increased maternal BMI was significantly related to elevated kynurenine, kynurenic acid and xanthurenic acid concentrations. Girls displayed elevated concentrations of 3-hydroxykynurenine, 3-hydroxyanthranilic acid and xanthurenic acid in their umbilical cord blood compared to boys.

We additionally found that maternal smoking status affected the umbilical cord blood concentrations of 3-hydroxykynurenine, quinolinic acid and picolinic acid. Particularly quinolinic acid and picolinic acid concentrations were reduced in the umbilical cord blood if the women smoked during the last four weeks of pregnancy. Moreover, infants who were delivered through caesarean sections had reduced umbilical cord blood concentrations of tryptophan and 3-hydroxyanthranilic acid, while kynurenine, 3-hydroxykynurenine and quinolinic acid were elevated. Maternal age was associated with statistically significant but minor elevations in kynurenine, 3-hydroxykynurenine, and quinolinic acid concentrations.

**Fig. 2:**
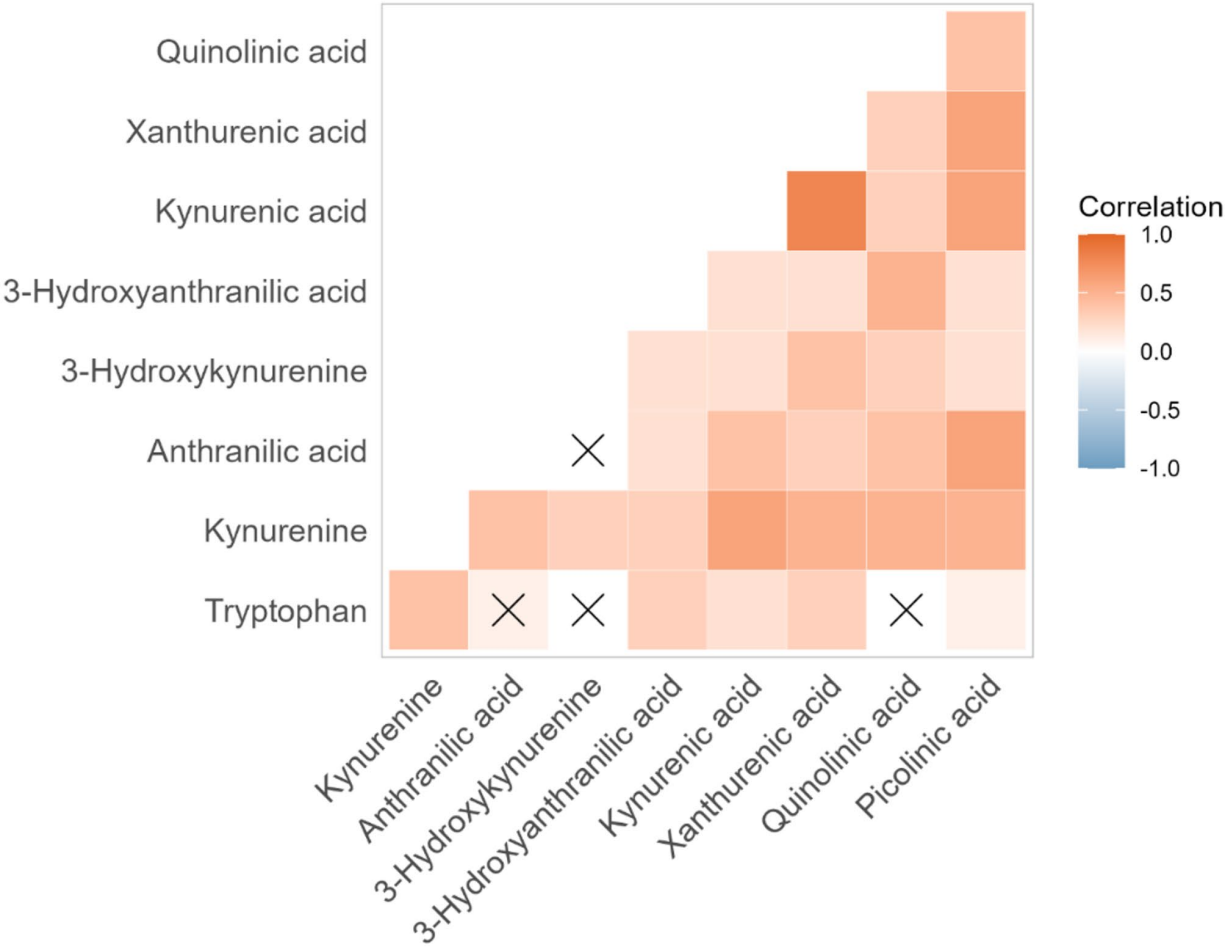
Correlations between kynurenine pathway (KP) metabolites concentrations in umbilical cord blood. The concentrations of most KP metabolites positively correlate with each other. The correlation between tryptophan and KP metabolites is weaker (lower R-values) and for anthranilic acid, 3-hydroxykynurenine and quinolinic acid not statistically significant, as denoted by the crosses (X)

In the full data set we observed a strong correlation between many of the different KP metabolites (Fig. 2). In particular, the first metabolite kynurenine, and the last metabolite picolinic acid correlated with all other metabolites (kynurenine: 0.271 < *R* < 0.500, picolinic acid: 0.166 < *R* < 0.592). The strongest correlation was observed between kynurenic acid and xanthurenic acid (*R* = 0.754). In comparison, the KP substrate tryptophan showed substantially lower correlation coefficients with all other KP metabolites.

### Preeclampsia

A full overview of the clinical descriptives of the preeclampsia population and their matched controls is given in Supplementary Table S1. The two groups showed no differences in median age (controls: 29.0 years, preeclampsia: 27.5 years), BMI (controls: 25.9 kg/m^2^, preeclampsia: 24.7 kg/m^2^), and gestational age (38.0 weeks for both). The population with preeclampsia displayed an increased percentage of caesarean sections (controls: 23.8%, preeclampsia: 42.1%) and reduced fetal birthweight (controls: 3282.5 g, preeclampsia: 2955.0 g). Within the preeclampsia group one patient suffered from FGR, three from diabetes and four from AIS.

**Fig. 3:**
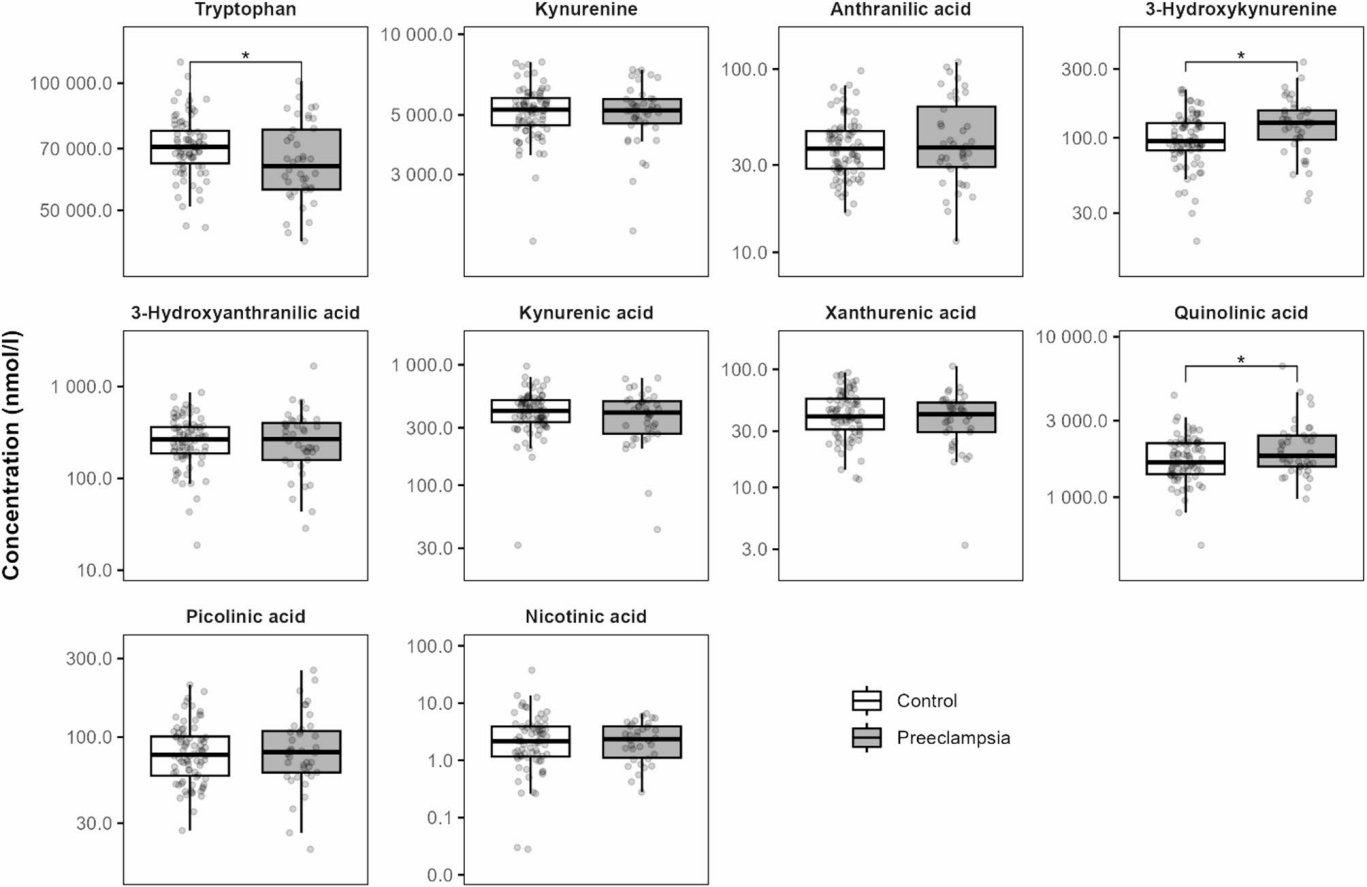
The effect of preeclampsia on KP metabolite concentrations in umbilical cord blood. * *P* < 0.05 calculated using a t-test on the natural log transformed data

Looking at the KP metabolites concentrations in umbilical cord blood, preeclampsia was associated with reduced concentrations of tryptophan, while the concentrations of 3-hydroxykynurenine and quinolinic acid were elevated (Fig. 3, Supplementary Table S2). A negative association between preeclampsia and tryptophan, as well as a positive association between preeclampsia and 3-hydroxykynurenine and quinolinic acid concentrations were confirmed in Model 1. After correction for confounders in Model 2, the effect of preeclampsia remained statistically significant for 3-hydroxykynurenine only. However, the tendencies for a negative association for tryptophan, and positive association for quinolinic acid with preeclampsia remained (Supplementary Table S3).

The correlation coefficients between KP metabolites seemed to be higher in preeclampsia than in controls (Supplementary Figure S7).

### Fetal growth restriction (FGR)

A full overview of the clinical descriptives of the FGR population and their matched controls is given in Supplementary Table S4. The two groups had a similar median age (controls: 30.0 years, FGR: 29.0 years), BMI (controls: 22.5 kg/m^2^, FGR: 22.2 kg/m^2^), and gestational age (38.0 weeks for both). Mothers of infants with FGR more often smoked during the last four weeks of pregnancy (controls: 4.5%, FGR: 25.0%) and were subjected to a higher percentage of caesarean sections (controls: 30.3%, FGR: 66.7%). Moreover, the population with FGR displayed a reduced median birthweight (controls: 3220.0 g, FGR: 2650.0 g) and placenta weight (controls: 545.0 g, FGR: 470.0), as well as an increased percentage of neonatal intensive care unit (NICU) admissions (controls: 18.5%, FGR: 62.5%). Within the FGR population one patient suffered from preeclampsia, two from GDM and three from AIS.

**Fig. 4:**
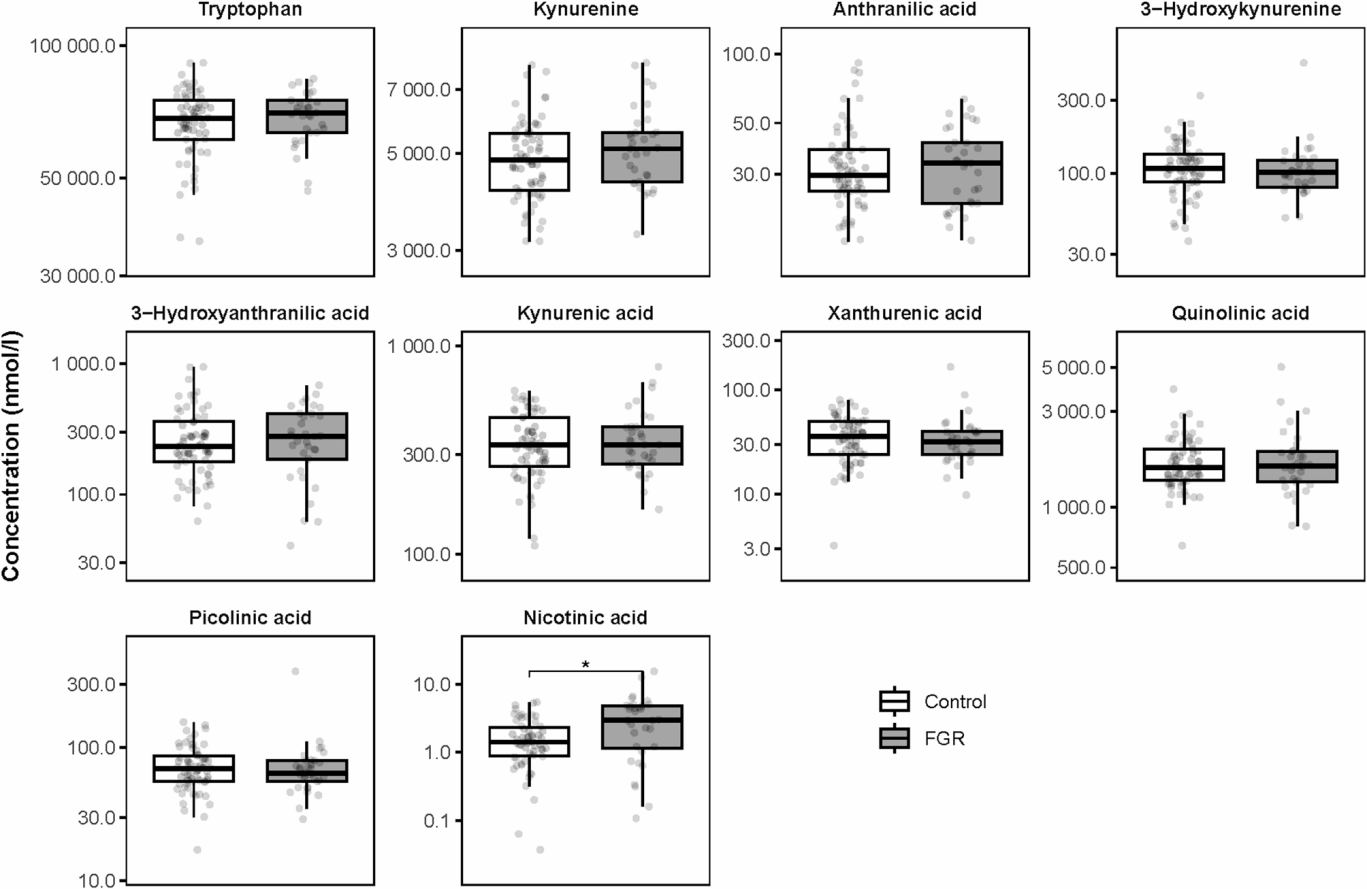
The effect of fetal growth restriction (FGR) on KP metabolite concentrations in umbilical cord blood. * *P* < 0.05 calculated using a t-test on the natural log transformed data

In the umbilical cord blood of FGR pregnancies, only nicotinic acid was statistically significantly elevated versus matched controls (Fig. 4, Supplementary Table S5). The fully adjusted Model 2 showed an even stronger positive association between FGR and nicotinic acid compared with the unadjusted Model 1. FGR was not associated with changes in the concentrations of any of the other KP metabolites (Supplementary Table S6).

Many of the correlations between KP metabolites that were observed in controls, particularly those with 3-hydroxykynurenine, were lost in FGR (Supplementary Figure S8).

### Pregestational diabetes mellitus (DM)

A full overview of the clinical descriptives of the DM population and their matched controls is given in Supplementary Table S7. The two groups both had a median BMI of 25.5 kg/m2 and gestational age of 39.0 weeks. Women with DM had a higher maternal age (controls: 29.0 (26.0, 33.0) kg/m^2^, DM: 31.5 (26.0, 34.0) kg/m^2^), and a higher rate of caesarean sections (controls: 22.6%, DM: 46.3%) and NICU admissions (controls: 8.4%, DM: 28.6%). Two women with DM also suffered from preeclampsia, while one was diagnosed with AIS.

**Fig. 5:**
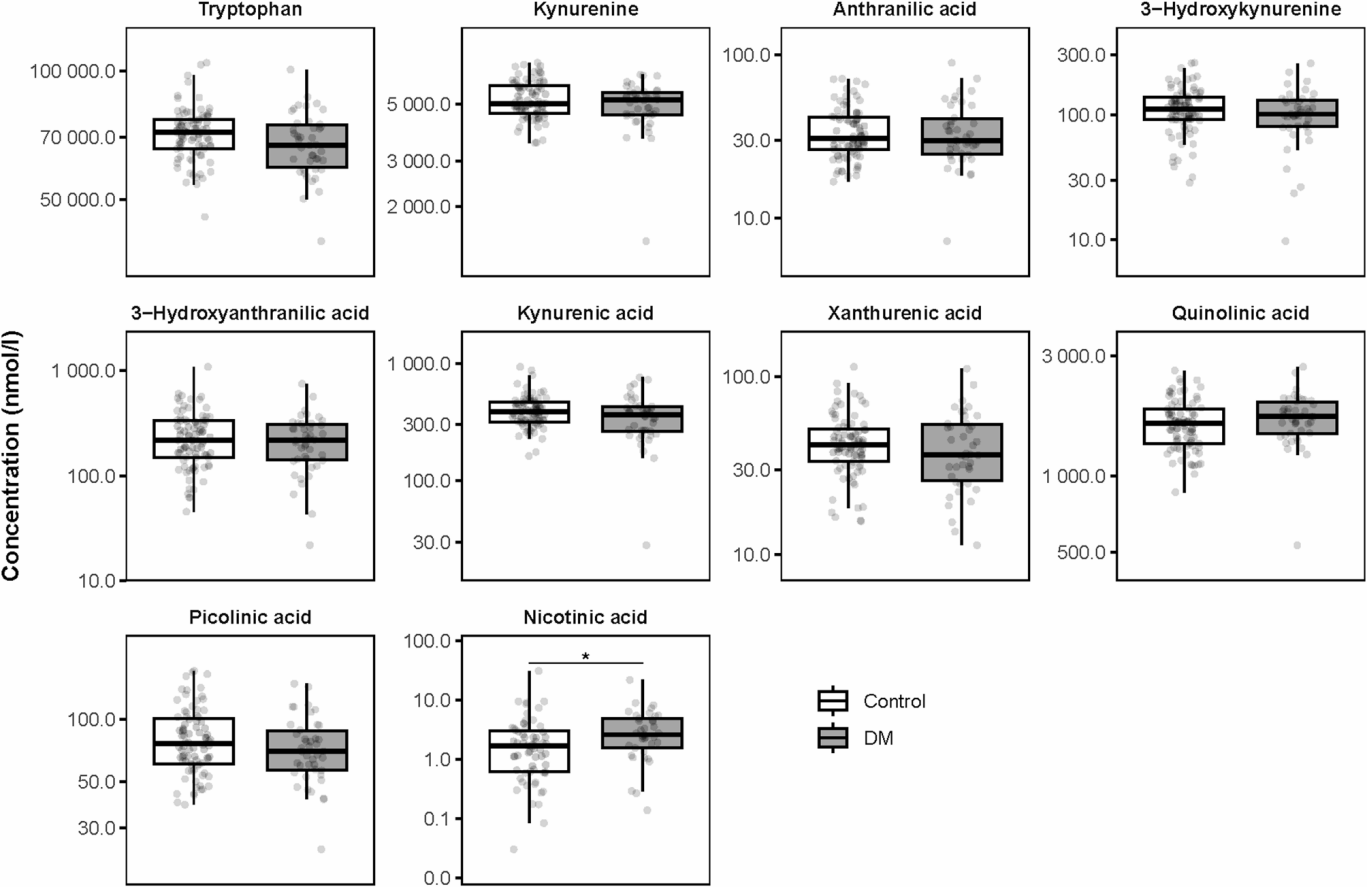
The effect of pregestational diabetes mellitus (DM) on KP metabolite concentrations in umbilical cord blood. * *P* < 0.05 calculated using a t-test on the natural log transformed data

The absolute concentration of nicotinic acid was elevated in the umbilical cord blood of pregnancies with DM (Fig. 5, Supplementary Table S8). This finding was confirmed by the positive association between DM and tryptophan in Model 1, which was strengthened after correction for multiple confounders in Model 2 (Additional file 1; Table S9). Although the crude data only showed a trend towards lower tryptophan concentrations in DM, both Model 1 and Model 2 displayed a statistically significant negative association between DM and tryptophan.

Correlations between KP metabolites, and particularly those with quinolinic acid were significantly reduced in DM (Supplementary Figure S9).

### Gestational diabetes mellitus (GDM)

A full overview of the clinical descriptives of the GDM population and their matched controls is given in Supplementary Table S10. The two groups had a similar median age (30.0 years for both), BMI (controls: 26.6 kg/m^2^, GDM: 26.7 kg/m^2^), and gestational age (39.0 weeks for both). The GDM population showed an increased rate of caesarean sections (controls: 28.3%, GDM: 43.3%). From the women with GDM, one also suffered from preeclampsia, two from FGR and four from AIS.

**Fig. 6:**
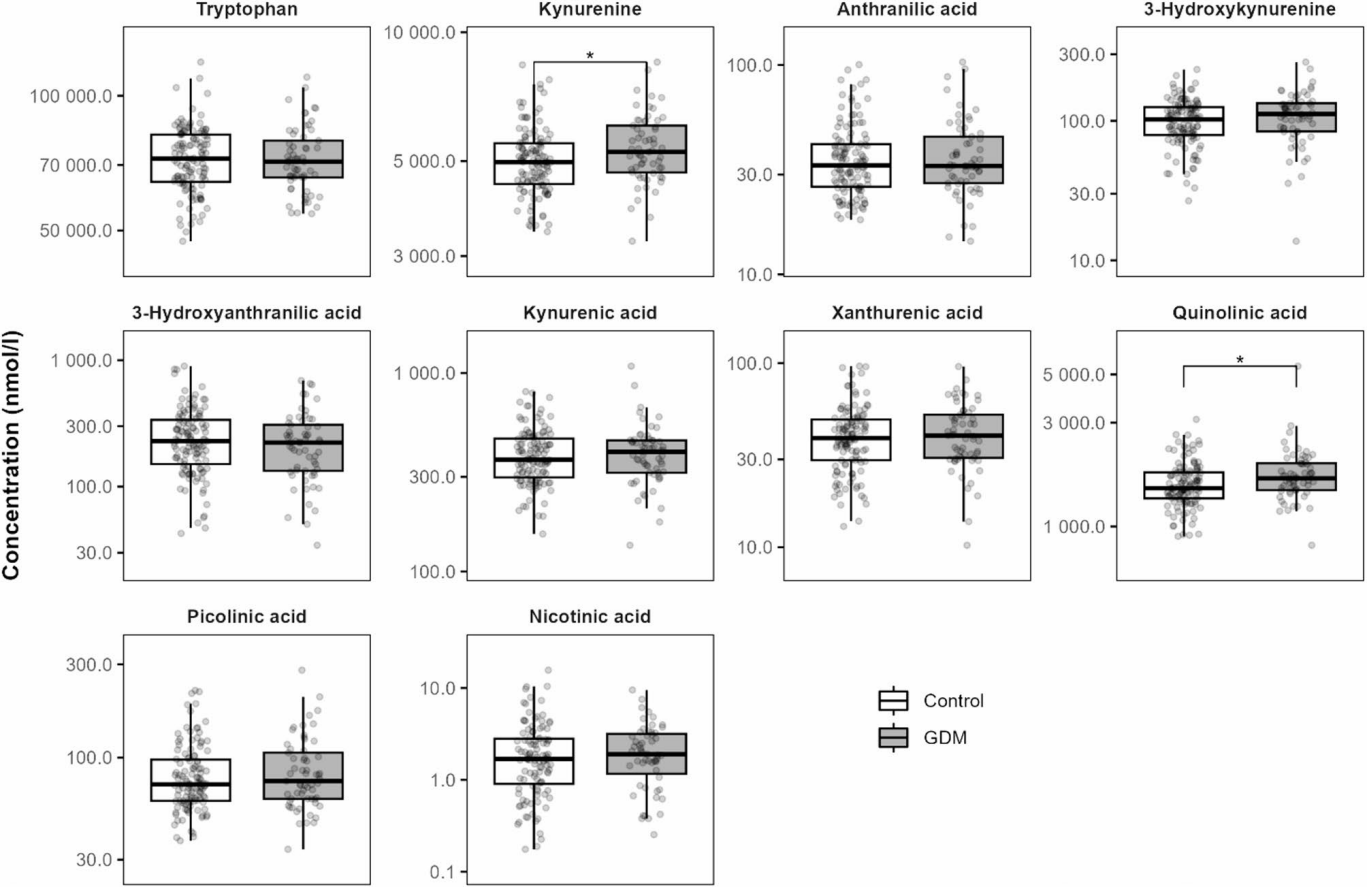
The effect of gestational diabetes mellitus (GDM) on KP metabolite concentrations in umbilical cord blood. * *P* < 0.05 calculated using a t-test on the natural log transformed data

Kynurenine and quinolinic acid concentrations were elevated in the umbilical cord blood with GDM (Fig. 6, Supplementary Table S11). Both Model 1 and Model 2 also showed positive associations between GDM and kynurenine as well as quinolinic acid (Supplementary Table S12).

Correlations between KP metabolites seemed similar in GDM and controls (Supplementary Figure S10).

### Amniotic infection syndrome (AIS)

A full overview of the clinical descriptives of the AIS population and their matched controls is given in Supplementary Table S13. The two groups had a similar median age (controls: 29.5 years, AIS: 29.0 years), BMI (controls: 23.7 kg/m^2^, AIS: 23.9 kg/m^2^), and gestational age (39.0 weeks for both). In the AIS group, four women also suffered from preeclampsia, three from FGR, four from GDM, and one from DM.

**Fig. 7:**
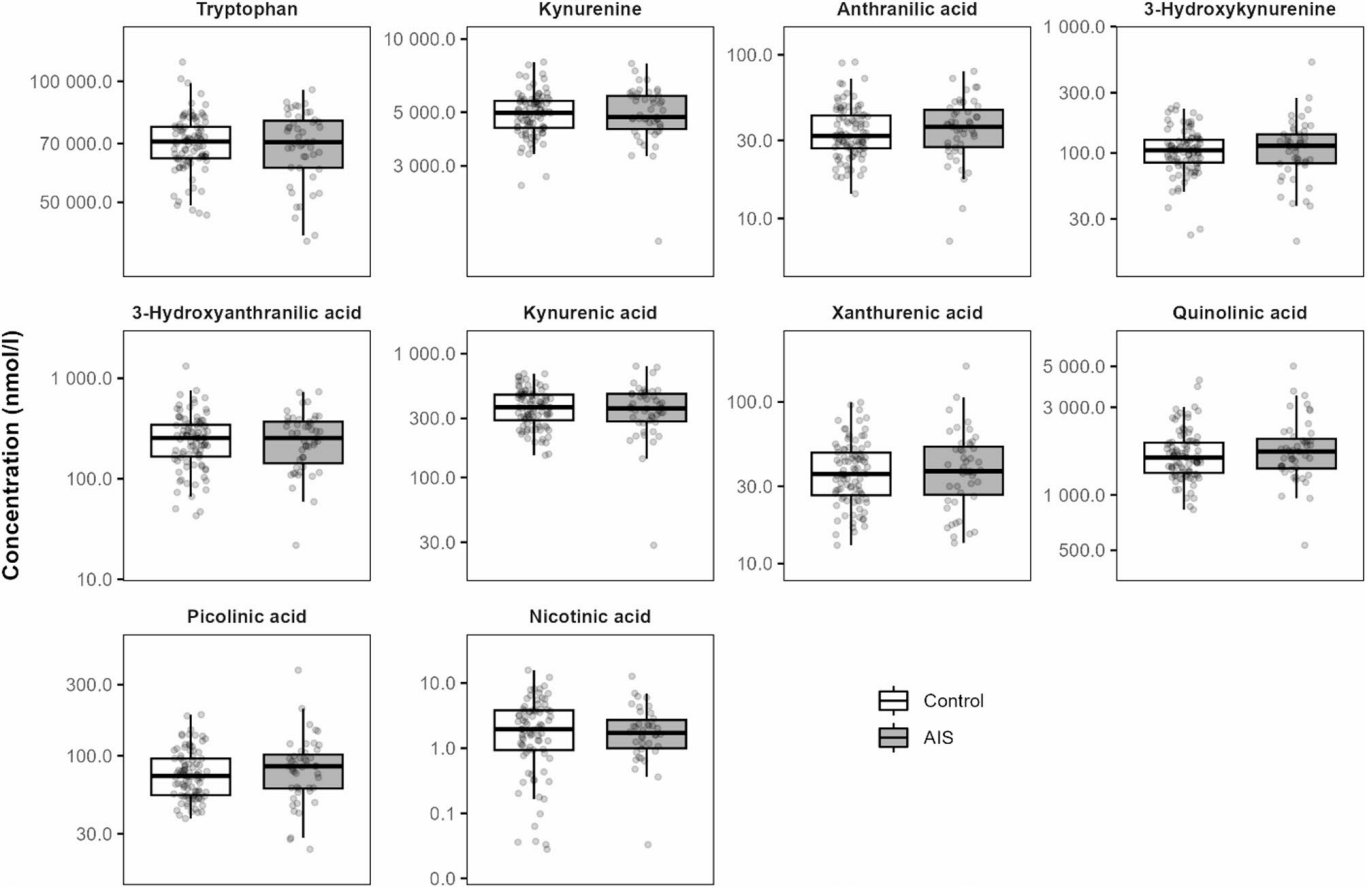
The effect of amniotic infection syndrome (AIS) on KP metabolite concentrations in umbilical cord blood. * *P* < 0.05 calculated using a t-test on the natural log transformed data

AIS was not associated with any changes in the concentrations of KP metabolites in umbilical cord blood (Fig. 7, Supplementary Tables S14 & S15).

The correlation coefficients between KP metabolite concentrations were also not significantly altered in AIS (Suppelementary Figure S11).

## Discussion

**Fig. 8:**
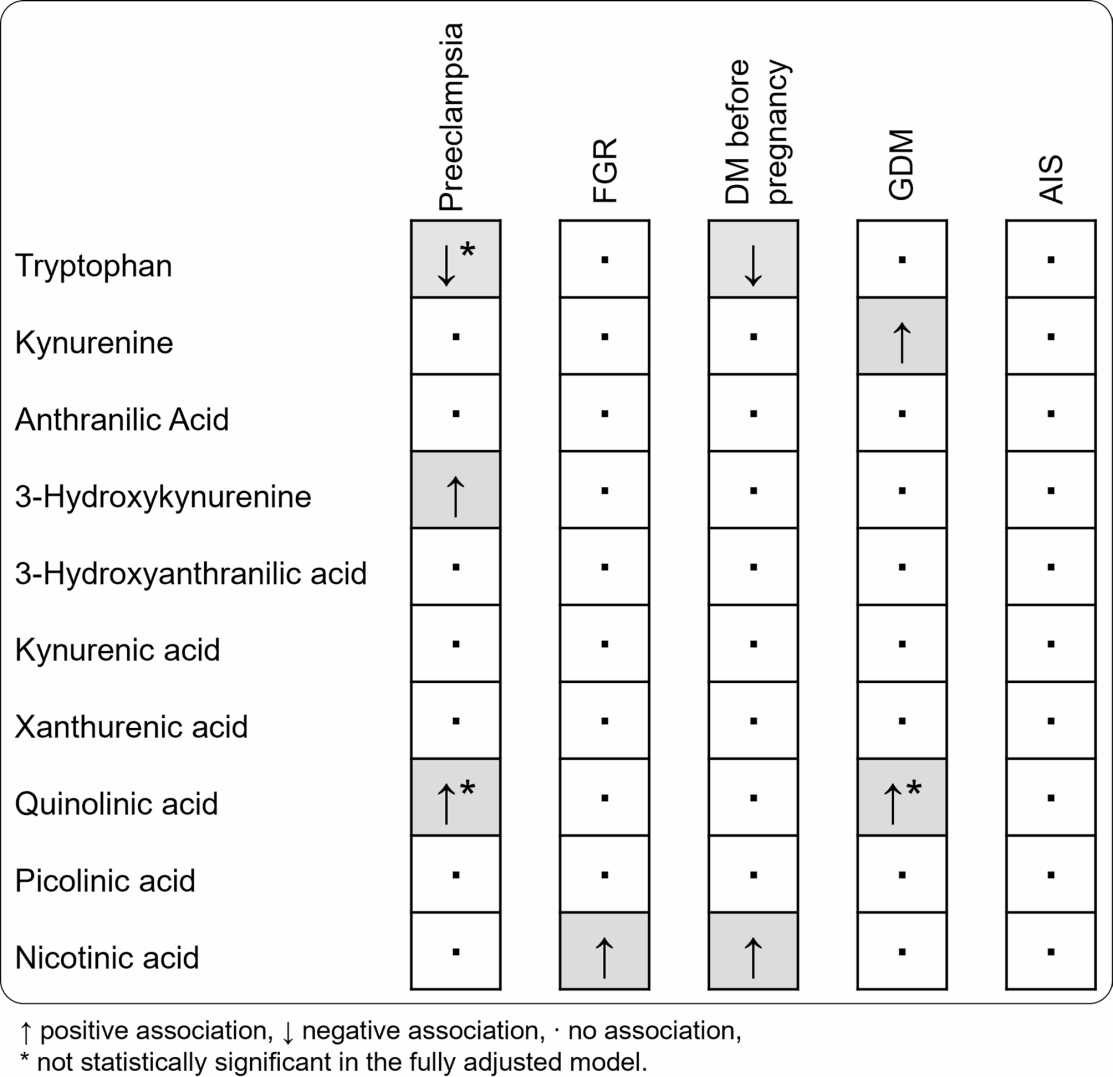
Summary of the associations between pregnancy complications and KP metabolites concentrations in umbilical cord blood. FGR denotes fetal growth restriction; DM, diabetes mellitus; GDM, gestational diabetes mellitus. ↓ indicates lower concentration; ↑ higher concentration; ∙ equal concentration compared with matched controls; * not statistically significant in the fully adjusted Model 2

Kynurenine pathway metabolites play multiple roles that are essential for a healthy pregnancy and fetal development, including modulation of the immune system, pro- and antioxidant effects and regulation of vascular development and tone [1]. Data from the current study demonstrate associations between the pregnancy-related complications preeclampsia, FGR and diabetes, and KP alterations in the umbilical cord blood which are summarized in Fig. 8. Tryptophan concentrations were negatively associated with both preeclampsia and DM. In contrast, all other associations were positive, indicating elevated levels of 3-hydroxykynurenine in preeclampsia, kynurenine in GDM, and nicotinic acid in FGR as well as DM. The positive associations of quinolinic acid with preeclampsia and GDM were no longer statistically significant in the fully adjusted models, suggesting gestational age as a major confounding factor. AIS did not affect the concentration of any KP metabolite in umbilical cord blood.

The distinct patterns of KP metabolite alterations across different pregnancy complications point to differential regulation and activation of the pathway. Overall, the combination of lower tryptophan and increased downstream metabolite concentrations in the fetal circulation may reflect enhanced KP activity in the fetus. Although this aligns with changes previously identified in maternal blood through our systematic review, it contrasts with findings in placental tissue [5]. Therefore, we believe these observations further support the hypothesis that physiological pregnancy depends on a tightly regulated KP balance, and that disturbances in either direction may contribute to adverse outcomes.

Changes in the concentrations of KP metabolites can originate from alterations in the maternal, placental, and/or fetal KP regulation. Generally, such changes in circulating KP metabolites, and particularly the kynurenine/tryptophan ratio, are used as a measure for placental IDO1 activity. Yet, data from this as well as earlier studies suggest that circulating KP metabolites do not necessarily reflect local KP changes in the placenta [5, 8]. Preeclampsia and FGR displayed distinct KP alterations in the umbilical cord blood, even though these disorders are often ascribed to have a similar pathophysiological origin in the placenta. Specifically, preeclampsia was associated with an elevated 3-hydroxykynurenine concentration in umbilical cord blood, while this was unaltered in FGR. The contradiction with Zhao et al. [30] who did not find an association between preeclampsia and concentrations of KP metabolites in umbilical cord blood, may be explained by our larger sample size and well-matched control cohort. 3-Hydroxykynurenine is one of the most potent free radical scavenging kynurenine metabolites, but it was also shown to mediate superoxide formation and endothelial cell apoptosis after angiotensin II infusion [31–33]. As women with preeclampsia are known to have increased levels of reactive oxygen species [34], we deem it likely that the elevate 3-hydroxykynurenine concentration in preeclampsia reflects the maternal inflammatory state. Such a maternal inflammatory state is usually not observed in FGR which may explain the distinct KP changes.

The elevated nicotinic acid concentration in umbilical cord blood with FGR in the absence of any other KP changes indicates a reduced consumption of nicotinic acid, potentially through an overall decrease in cellular metabolism; less optimal conversion of nicotinic acid into nicotinic acid mononucleotide. Nicotinic acid mononucleotide can be formed from quinolinic acid and is a precursor for nicotinamide adenine dinucleotide (NAD) biosynthesis generating key metabolites NAD^+^, NADP, NADH and NADPH, which are essential in virtually every aspect of cellular metabolism, particularly mitochondria, and which are often altered in conditions of oxidative stress [35]. The elevated nicotinic acid concentration may thus be a consequence of the smaller fetus, but it could also provide a protective mechanism for fetal development. Nicotinic acid has positive effects on lipid metabolism and regulates the transport and metabolism of glucose by stimulating its flux through the pentose phosphate pathway [36]. The latter is particularly interesting since we also found an enhanced nicotinic acid concentration in the umbilical cord blood in pregnancies with DM.

In pregnancies with DM the increased formation of nicotinic acid may provide a mechanism that protects the fetus from too high glucose concentrations. This differs from pregnancies with GDM where nicotinic acid was not altered in umbilical cord blood. Instead, in GDM we found an increased concentration of kynurenine, which is in concordance with the increased kynurenine concentrations that were identified through metabolomics in maternal blood already in the first trimester [37–39]. The distinct KP metabolite alterations in umbilical cord blood between DM and GDM have not been presented before and warrant further research.

AIS was not associated with changes in the concentrations of KP metabolites. Although several studies reported an association between inflammation and changes in KP metabolism [29, 40, 41], studies on KP metabolism in intrauterine and neonatal infection, which may result from intrauterine infection, are scarce. In a small sample, Manuelpillai et al. [42] found higher concentrations of kynurenine and quinolinic acid in the umbilical cord blood of a small sample of preterm fetuses exposed to intrauterine infections compared to those without infection. Absence of KP changes in AIS may result from the fact that compared with disorders like GDM, FGR and preeclampsia, AIS is an acute condition that often rapidly induces (preterm) termination of pregnancy, potentially before it can have an effect on umbilical cord blood concentrations of KP metabolites. In this context, acute immune activation during pregnancy also had only short-lasting effects on cerebral KP metabolism in an experimental mouse model [43].

The concentration of tryptophan in umbilical cord we report here is approximately twice as high as the average concentration in maternal blood during healthy pregnancy [5]. The concentration of kynurenine is even almost five times as high in umbilical versus maternal blood [5]. This suggests not only active transfer of tryptophan from mother to fetus, but also enhanced metabolism of tryptophan into kynurenine. Although the placenta facilitates transfer of tryptophan from the maternal to the fetal circulation, our previous results suggest that placental KP activity unlikely contributes to the enhanced kynurenine concentrations in the fetal circulation [8]. This means that the high concentration gradient of kynurenine in the fetal circulation likely arises from KP activity in the organs of the fetus or significant placental transfer of kynurenine from the mother to the fetus. Moreover, the concentrations of kynurenic acid and xanthurenic acid are highly correlated in umbilical cord blood, likely because they are both formed by the same enzyme; kynurenine aminotransferase.

### Strengths and limitations

This study is the first to investigate the associations between multiple pregnancy complications and KP concentrations in umbilical cord blood in a large cohort. The well-matched cohort diminishes confounding bias and improves the internal validity, although the external validity may be limited due to the non-random selection of matched controls a priori. In our data BMI, gestational age and fetal sex showed strong associations, and smoking a tendency towards an association with many KP metabolites (Supplementary Figures S1-S4). Therefore, we also corrected for these potential confounders in the linear regression models. However, it should be noted that we observed an additional effect of mode of delivery on KP metabolites. Caesarean section was associated with elevated tryptophan and 3-hydroxyanthranilic acid concentrations, as well as decreased 3-hydroxykynurenine and quinolinic acid concentrations (Supplementary Figure S5). However, since mode of delivery was not an idependent variable, we could not include this in our regression models, bringing in some level of uncertainty regarding the contribution of mode of delivery to our results.

It should also be noted that this study identified a large variation, greater than 10-fold differences, in the concentrations of most KP metabolites. This intragroup variation was significantly larger than the intergroup differences, adding uncertainty towards the relevance of the intergroup differences.

## Conclusions

The kynurenine pathway (KP) plays a critical role in maintaining a healthy pregnancy. This study demonstrates that preeclampsia, fetal growth restriction (FGR), diabetes, and amniotic infection syndrome (AIS) are each associated with distinct alterations in KP metabolite concentrations in umbilical cord blood. While it remains unclear whether these alterations are causal or a consequence of the underlying pathology, they are likely to influence fetal development and may have lasting implications for the infant’s health after birth. In particular because KP metabolites are important for many physiological systems in the body and have crucial roles in neuronal, pulmonary and cardiovascular development and function [44–50]. Future research is warranted to determine whether the identified KP changes persist postnatally, and whether they contribute to fetal programming, potentially affecting long-term development, and disease susceptibility in the offspring.

## Electronic supplementary material

Below is the link to the electronic supplementary material.

Supplementary Material 1

## Data Availability

The datasets used and analysed during the current study are available from the corresponding author on reasonable request.

## Abbreviations

AIS: Amniotic infection syndrome
BMI: Body mass index
DM: Diabetes mellitus before pregnancy
FGR: Fetal growth restriction
GDM: Gestational diabetes mellitus
IDO: Indoleamine 2,3-dioxygenase
KP: Kynurenine pathway
LC-MS/MS: Liquid chromatography tandem mass spectrometry
NAD: Nicotinamide adenine dinucleotide
NICU: Neonatal intensive care unit
SNiP: Survey of neonates in Pomerania
