# Supplementary material for: Pregnancy complications affect kynurenine pathway metabolite concentrations in umbilical cord blood
Source: Reprod Biol Endocrinol. 2025 Jul 21;23:105. doi: 10.1186/s12958-025-01436-6 (PMC12278539; doi:10.1186/s12958-025-01436-6)
Supplement: Supplementary file 1 — Supplementary Material 1 [file 12958_2025_1436_MOESM1_ESM.docx]

Additional file 1

Belonging to *Pregnancy complications affect kynurenine pathway metabolite concentrations in umbilical cord blood* by Broekhuizen et al.

**Supplemental Table S1.** Clinical descriptives of the preeclampsia cohort and their matched controls.

| **Variable** | **Control (n = 80)** | **Preeclampsia (n = 40)** | **P-value** |
| --- | --- | --- | --- |
| **Age mom** | 29.0 (27.0, 32.0) | 27.5 (25.0, 30.2) | 0.149 |
| **BMI (kg/m^2^)** | 24.7 (22.6, 28.0) | 25.9 (22.8, 31.9) | 0.177 |
| **Smoking** |  |  | 0.44 |
| never | 31 (39.7%) | 15 (37.5%) |  |
| former | 38 (48.7%) | 23 (57.5%) |  |
| last 4 weeks of pregnancy | 9 (11.5%) | 2 (5.0%) |  |
| **Cesarean section** | 19 (23.8%) | 16 (42.1%) | **0.041** |
| **Gestational age (weeks)** | 38.0 (36.0, 39.2) | 38.0 (35.0, 39.0) | 0.382 |
| **Sex girl** | 38 (47.5%) | 19 (47.5%) | 1 |
| **Birth weight (grams)** | 3282.5 (2807.5, 3592.5) | 2955.0 (2393.8, 3495.0) | **0.045** |
| **Placenta weight (grams)** | 570.0 (470.0, 675.0) | 540.0 (410.0, 620.0) | 0.144 |
| **NICU admission** | 21 (26.6%) | 17 (42.5%) | 0.079 |
| **Preeclampsia** | 0 (0.0%) | 40 (100.0%) |  |
| **FGR** | 0 (0.0%) | 1 (2.5%) |  |
| **GDM** | 0 (0.0%) | 1 (2.5%) |  |
| **DM** | 0 (0.0%) | 2 (5.0%) |  |
| **AIS** | 0 (0.0%) | 4 (10.0%) |  |

Abbreviations: BMI, body mass index; NICU, neonatal intensive care unit; FGR, fetal growth restriction; GDM, gestational diabetes mellitus; DM, diabetes mellitus before pregnancy; AIS, amniotic infection syndrome.

**Supplemental Table S2.** Umbilical cord blood KP metabolite concentrations in the preeclampsia (PE) cohort and their matched controls.

| **Variable** | **Control (n = 80)** | **Preeclampsia (n = 40)** | **P value** | **Adj. P** |
| --- | --- | --- | --- | --- |
| Tryptophan | 70628 (64595, 77157) | 63661 (56006, 77643) | **0.0286** | 0.11 |
| Kynurenine | 5235 (4577, 5787) | 5202 (4652, 5734) | 0.854 | 0.854 |
| Anthranilic acid | 36 (28, 45) | 37 (29, 62) | 0.471 | 0.785 |
| 3-Hydroxykynurenine | 94 (81, 126) | 127 (97, 155) | **0.0196** | 0.11 |
| 3-Hydroxyanthranilic acid | 264 (185, 361) | 268 (157, 400) | 0.837 | 0.854 |
| Kynurenic acid | 413 (333, 507) | 400 (267, 497) | 0.259 | 0.638 |
| Xanthurenic acid | 39 (30, 56) | 41 (29, 52) | 0.319 | 0.638 |
| Quinolinic acid | 1651 (1392, 2175) | 1811 (1553, 2428) | **0.033** | 0.11 |
| Picolinic acid | 78 (58, 100) | 80 (60, 108) | 0.678 | 0.854 |
| Nicotinic acid | 2 (1, 3) | 2 (1, 3) | 0.795 | 0.854 |
| Concentrations are depicted in nmol/l as median (Q1, Q3). The P values were calculated on the natural log transformed data using t-tests adjusted with BH correction (adj. P). | | | | |

**Supplemental Table S3.** Linear regression table depicting the effect estimates of preeclampsia on the umbilical cord blood concentrations of KP metabolites. Model 1 is only adjusted for the preeclampsia. Model 2 is additionally adjusted for fetal sex, as well as maternal BMI, gestational age, and smoking status.

|  | **Model 1** | | **Model 2** | |
| --- | --- | --- | --- | --- |
| **Dependent** | **Estimate** | **95% CI** | **Estimate** | **95% CI** |
| Tryptophan | **-0.07 *** | **[-0.14, -0.00]** | -0.06 | [-0.13, 0.01] |
| Kynurenine | 0.02 | [-0.06, 0.10] | -0.01 | [-0.09, 0.07] |
| Anthranilic Acid | 0.11 | [-0.06, 0.27] | 0.06 | [-0.10, 0.21] |
| 3-Hydroxy kynurenine | **0.24 **** | **[0.06, 0.41]** | **0.19 *** | **[0.01, 0.36]** |
| 3-Hydroxy anthranilic acid | -0.03 | [-0.28, 0.22] | -0.09 | [-0.34, 0.16] |
| Kynurenic acid | -0.06 | [-0.19, 0.08] | -0.08 | [-0.22, 0.06] |
| Xanthurenic acid | -0.05 | [-0.22, 0.13] | -0.08 | [-0.26, 0.09] |
| Quinolinic acid | **0.16 *** | **[0.04, 0.29]** | 0.10 | [-0.02, 0.21] |
| Picolinic acid | 0.09 | [-0.07, 0.25] | 0.04 | [-0.13, 0.21] |
| Nicotinic acid | -0.11 | [-0.50, 0.29] | -0.14 | [-0.55, 0.27] |
| All continuous predictors are mean-centered and scaled by 1 standard deviation. The dependent variables are in the log conversed value and N = 115 for all except for nicotinic acid N = 100. *** p < 0.001; ** p < 0.01; * p < 0.05. | | | | |

**Supplemental Table S4.** Clinical descriptives of the fetal growth restriction (FGR) cohort and their matched controls.

| **Variable** | **Control (n = 66)** | **FGR (n = 33)** | **P-value** |
| --- | --- | --- | --- |
| **Age mom** | 30.0 (27.0, 34.0) | 29.0 (27.0, 35.0) | 0.78 |
| **BMI (kg/m^2^)** | 22.5 (20.1, 26.3) | 22.2 (20.4, 26.3) | 0.722 |
| **Smoking** |  |  | **0.01** |
| never | 26 (39.4%) | 11 (34.4%) |  |
| former | 37 (56.1%) | 13 (40.6%) |  |
| last 4 weeks of pregnancy | 3 (4.5%) | 8 (25.0%) |  |
| **Cesarean section** | 20 (30.3%) | 22 (66.7%) | **< 0.001** |
| **Gestational age (weeks)** | 38.0 (37.0, 40.0) | 38.0 (36.0, 40.0) | 0.584 |
| **Sex girl** | 41 (62.1%) | 21 (63.6%) | 0.883 |
| **Birth weight (grams)** | 3220.0 (2886.2, 3656.2) | 2650.0 (2330.0, 2910.0) | **< 0.001** |
| **Placenta weight (grams)** | 545.0 (485.0, 620.0) | 470.0 (415.0, 505.0) | **< 0.001** |
| **NICU admission** | 12 (18.5%) | 20 (62.5%) | **< 0.001** |
| **Preeclampsia** | 0 (0.0%) | 1 (3.0%) |  |
| **FGR** | 0 (0.0%) | 33 (100.0%) |  |
| **GDM** | 0 (0.0%) | 2 (6.1%) |  |
| **DM** | 0 (0.0%) | 0 (0.0%) |  |
| **AIS** | 0 (0.0%) | 3 (9.1%) |  |

Abbreviations: BMI, body mass index; NICU, neonatal intensive care unit; FGR, fetal growth restriction; GDM, gestational diabetes mellitus; DM, diabetes mellitus before pregnancy; AIS, amniotic infection syndrome.

**Supplemental Table S5.** Umbilical cord blood KP metabolite concentrations in the fetal growth restriction (FGR) cohort and their matched controls.

| **Variable** | **Control (n = 66)** | **FGR (n = 33)** | **P value** | **Adj. P** |
| --- | --- | --- | --- | --- |
| Tryptophan | 68260 (61304, 75124) | 70404 (63478, 75217) | 0.272 | 0.907 |
| Kynurenine | 4823 (4114, 5537) | 5114 (4298, 5579) | 0.166 | 0.83 |
| Anthranilic acid | 29 (25, 38) | 33 (22, 41) | 0.962 | 0.962 |
| 3-Hydroxykynurenine | 108 (88, 133) | 101 (81, 121) | 0.831 | 0.962 |
| 3-Hydroxyanthranilic acid | 232 (178, 360) | 277 (186, 413) | 0.953 | 0.962 |
| Kynurenic acid | 333 (264, 454) | 335 (271, 408) | 0.659 | 0.962 |
| Xanthurenic acid | 35 (24, 49) | 32 (23, 40) | 0.93 | 0.962 |
| Quinolinic acid | 1576 (1362, 1945) | 1606 (1346, 1901) | 0.863 | 0.962 |
| Picolinic acid | 69 (55, 86) | 64 (55, 79) | 0.861 | 0.962 |
| Nicotinic acid | 1 (0, 2) | 3 (1, 4) | **0.0354** | 0.354 |
| Concentrations are depicted in nmol/l as median (Q1, Q3). The P values were calculated using t-tests adjusted with BH correction (adj. P) on the natural log transformed data | | | | |

**Supplemental Table S6.** Linear regression table depicting the effect estimates of fetal growth restriction (FGR) on the umbilical cord blood concentrations of KP metabolites. Model 1 is only adjusted for the preeclampsia. Model 2 is additionally adjusted for fetal sex, as well as maternal BMI, gestational age, and smoking status.

|  | **Model 1** | | **Model 2** | |
| --- | --- | --- | --- | --- |
| **Dependent** | **Estimate** | **95% CI** | **Estimate** | **95% CI** |
| Tryptophan | 0.04 | [-0.03, 0.11] | 0.05 | [-0.03, 0.12] |
| Kynurenine | 0.04 | [-0.04, 0.13] | 0.06 | [-0.04, 0.15] |
| Anthranilic Acid | -0.03 | [-0.20, 0.14] | 0.02 | [-0.16, 0.20] |
| 3-Hydroxy kynurenine | -0.09 | [-0.24, 0.07] | -0.10 | [-0.26, 0.07] |
| 3-Hydroxy anthranilic acid | -0.05 | [-0.31, 0.21] | -0.05 | [-0.30, 0.20] |
| Kynurenic acid | -0.01 | [-0.16, 0.14] | 0.01 | [-0.14, 0.17] |
| Xanthurenic acid | -0.10 | [-0.29, 0.10] | -0.10 | [-0.32, 0.11] |
| Quinolinic acid | -0.04 | [-0.16, 0.09] | -0.03 | [-0.15, 0.10] |
| Picolinic acid | -0.09 | [-0.24, 0.06] | -0.06 | [-0.22, 0.10] |
| Nicotinic acid | **0.52 *** | **[0.05, 0.98]** | **0.81 **** | **[0.33, 1.28]** |
| All continuous predictors are mean-centered and scaled by 1 standard deviation. The dependent variables are in the log conversed value and N = 97 for all except for nicotinic acid N = 88. *** p < 0.001; ** p < 0.01; * p < 0.05. | | | | |

**Supplemental Table S7.** Clinical descriptives of the diabetes mellitus before pregnancy (DM) cohort and their matched controls.

| **Variable** | **Control (n = 84)** | **DM (n = 42)** | **P-value** |
| --- | --- | --- | --- |
| **Age mom** | 29.0 (26.0, 33.0) | 31.5 (29.0, 34.0) | **0.027** |
| **BMI (kg/m^2^)** | 25.5 (23.2, 29.3) | 25.5 (23.3, 30.9) | 0.557 |
| **Smoking** |  |  | 0.877 |
| never | 27 (32.9%) | 15 (36.6%) |  |
| former | 44 (53.7%) | 20 (48.8%) |  |
| last 4 weeks of pregnancy | 11 (13.4%) | 6 (14.6%) |  |
| **Cesarean section** | 19 (22.6%) | 19 (46.3%) | **0.007** |
| **Gestational age (weeks)** | 39.0 (38.0, 39.0) | 39.0 (38.0, 39.0) | 0.454 |
| **Sex girl** | 40 (47.6%) | 20 (47.6%) | 1 |
| **Birth weight (grams)** | 3485.0 (3175.0, 3832.5) | 3390.0 (3150.0, 3745.0) | 0.278 |
| **Placenta weight (grams)** | 610.0 (530.0, 695.0) | 605.0 (535.0, 650.0) | 0.388 |
| **NICU admission** | 7 (8.4%) | 12 (28.6%) | **0.003** |
| **Preeclampsia** | 0 (0.0%) | 2 (4.8%) |  |
| **FGR** | 0 (0.0%) | 0 (0.0%) |  |
| **AIS** | 0 (0.0%) | 1 (2.4%) |  |

Abbreviations: BMI, body mass index; NICU, neonatal intensive care unit; FGR, fetal growth restriction; GDM, gestational diabetes mellitus; DM, diabetes mellitus before pregnancy; AIS, amniotic infection syndrome.

**Supplemental Table S8.** Umbilical cord blood KP metabolite concentrations in the diabetes mellitus before pregnancy (DM) cohort and their matched controls.

| **Variable** | **Control (n = 84)** | **DM (n = 42)** | **P value** | **Adj. P** |
| --- | --- | --- | --- | --- |
| Tryptophan | 71978 (65852, 77134) | 67166 (59600, 74755) | 0.06 | 0.27 |
| Kynurenine | 5009 (4596, 5903) | 5169 (4523, 5547) | 0.14 | 0.29 |
| Anthranilic acid | 30 (26, 41) | 29 (24, 40) | 0.79 | 0.79 |
| 3-Hydroxykynurenine | 110 (91, 137) | 101 (80, 130) | 0.23 | 0.38 |
| 3-Hydroxyanthranilic acid | 218 (149, 336) | 219 (141, 310) | 0.41 | 0.45 |
| Kynurenic acid | 386 (318, 469) | 364 (264, 425) | 0.08 | 0.27 |
| Xanthurenic acid | 41 (33, 50) | 36 (25, 53) | 0.30 | 0.38 |
| Quinolinic acid | 1622 (1347, 1845) | 1722 (1479, 1965) | 0.27 | 0.38 |
| Picolinic acid | 75 (60, 100) | 69 (56, 87) | 0.15 | 0.29 |
| Nicotinic acid | 1 (0, 2) | 2 (1, 4) | **0.01** | 0.14 |
| Concentrations are depicted in nmol/l as median (Q1, Q3). The P values were calculated using t-tests adjusted with BH correction (adj. P) on the natural log transformed data. | | | | |

**Supplemental Table S9.** Linear regression table depicting the effect estimates of diabetes mellitus before pregnancy (DM) on the umbilical cord blood concentrations of KP metabolites. Model 1 is only adjusted for the preeclampsia. Model 2 is additionally adjusted for fetal sex, as well as maternal BMI, gestational age, and smoking status.

|  | **Model 1** | | **Model 2** | |
| --- | --- | --- | --- | --- |
| **Dependent** | **Estimate** | **95% CI** | **Estimate** | **95% CI** |
| Tryptophan | **-0.06 *** | **[-0.11, -0.00]** | **-0.06 *** | **[-0.12, -0.00]** |
| Kynurenine | -0.03 | [-0.09, 0.04] | -0.03 | [-0.10, 0.03] |
| Anthranilic Acid | -0.02 | [-0.16, 0.12] | -0.03 | [-0.16, 0.11] |
| 3-Hydroxy kynurenine | 0.01 | [-0.16, 0.17] | 0.01 | [-0.15, 0.18] |
| 3-Hydroxy anthranilic acid | -0.10 | [-0.33, 0.13] | -0.12 | [-0.35, 0.12] |
| Kynurenic acid | -0.06 | [-0.18, 0.06] | -0.08 | [-0.21, 0.04] |
| Xanthurenic acid | -0.02 | [-0.18, 0.14] | -0.03 | [-0.20, 0.13] |
| Quinolinic acid | 0.08 | [-0.01, 0.16] | 0.07 | [-0.01, 0.15] |
| Picolinic acid | -0.08 | [-0.21, 0.06] | -0.09 | [-0.23, 0.05] |
| Nicotinic acid | **0.49 *** | **[0.00, 0.97]** | **0.59 *** | **[0.08, 1.10]** |
| All continuous predictors are mean-centered and scaled by 1 standard deviation. The dependent variables are in the log conversed value and N = 122 for all except for nicotinic acid N = 98. *** p < 0.001; ** p < 0.01; * p < 0.05. | | | | |

**Supplemental Table S10.** Clinical descriptives of the gestational diabetes mellitus (GDM) cohort and their matched controls.

| **Variable** | **Control (n = 122)** | **GDM (n = 61)** | **P-value** |
| --- | --- | --- | --- |
| **Age mom** | 30.0 (27.0, 32.0) | 30.0 (27.0, 34.0) | 0.213 |
| **BMI (kg/m^2^)** | 26.6 (23.4, 29.7) | 26.7 (23.7, 31.2) | 0.446 |
| **Smoking** |  |  | 0.999 |
| never | 47 (39.2%) | 24 (39.3%) |  |
| former | 61 (50.8%) | 31 (50.8%) |  |
| last 4 weeks of pregnancy | 12 (10.0%) | 6 (9.8%) |  |
| **Cesarean section** | 34 (28.3%) | 26 (43.3%) | **0.044** |
| **Gestational age (weeks)** | 39.0 (38.0, 40.0) | 39.0 (38.0, 40.0) | 0.605 |
| **Sex girl** | 46 (37.7%) | 23 (37.7%) | 1 |
| **Birth weight (grams)** | 3432.5 (3140.0, 3850.0) | 3570.0 (3210.0, 3820.0) | 0.604 |
| **Placenta weight (grams)** | 555.0 (500.0, 640.0) | 612.5 (507.5, 720.0) | 0.076 |
| **NICU admission** | 15 (12.4%) | 11 (18.0%) | 0.305 |
| **Preeclampsia** | 0 (0.0%) | 1 (1.6%) | 0.156 |
| **FGR** | 0 (0.0%) | 2 (3.3%) | **0.044** |
| **AIS** | 0 (0.0%) | 4 (6.6%) | **0.004** |

Abbreviations: BMI, body mass index; NICU, neonatal intensive care unit; FGR, fetal growth restriction; GDM, gestational diabetes mellitus; DM, diabetes mellitus before pregnancy; AIS, amniotic infection syndrome.

**Supplemental Table S11.** Umbilical cord blood KP metabolite concentrations in the gestational diabetes mellitus (GDM) cohort and their matched controls.

| **Variable** | **Control (n = 122)** | **GDM (n = 61)** | **P value** | **Adj. P** |
| --- | --- | --- | --- | --- |
| Tryptophan | 72312 (64191, 81850) | 71218 (65644, 79384) | 0.91 | 0.91 |
| Kynurenine | 4974 (4421, 5506) | 5255 (4708, 6058) | **0.02** | 0.08 |
| Anthranilic acid | 33 (26, 41) | 33 (27, 45) | 0.87 | 0.91 |
| 3-Hydroxykynurenine | 102 (79, 125) | 111 (83, 133) | 0.29 | 0.78 |
| 3-Hydroxyanthranilic acid | 229 (151, 336) | 222 (133, 308) | 0.31 | 0.78 |
| Kynurenic acid | 366 (298, 467) | 401 (314, 457) | 0.68 | 0.85 |
| Xanthurenic acid | 39 (29, 49) | 40 (30, 52) | 0.57 | 0.82 |
| Quinolinic acid | 1499 (1345, 1772) | 1664 (1469, 1951) | **0.01** | 0.08 |
| Picolinic acid | 72 (60, 97) | 75 (61, 105) | 0.46 | 0.82 |
| Nicotinic acid | 1 (0, 2) | 1 (1, 3) | 0.52 | 0.82 |
| Concentrations are depicted in nmol/l as median (Q1, Q3). The P values were calculated using t-tests adjusted with BH correction (adj. P) on the natural log transformed data. | | | | |

**Supplemental Table S12.** Linear regression table depicting the effect of gestational diabetes mellitus (GDM) on the umbilical cord blood concentrations of KP metabolites. Model 1 is only adjusted for the preeclampsia. Model 2 is additionally adjusted for fetal sex, as well as maternal BMI, gestational age, and smoking status.

|  | **Model 1** | | **Model 2** | |
| --- | --- | --- | --- | --- |
| **Dependent** | **Estimate** | **95% CI** | **Estimate** | **95% CI** |
| Tryptophan | 0.00 | [-0.05, 0.05] | -0.01 | [-0.06, 0.05] |
| Kynurenine | **0.07 *** | **[0.01, 0.12]** | **0.06 *** | **[0.00, 0.12]** |
| Anthranilic Acid | 0.00 | [-0.12, 0.12] | 0.01 | [-0.11, 0.13] |
| 3-Hydroxy kynurenine | 0.11 | [-0.02, 0.23] | 0.10 | [-0.03, 0.22] |
| 3-Hydroxy anthranilic acid | -0.11 | [-0.30, 0.09] | -0.13 | [-0.32, 0.05] |
| Kynurenic acid | 0.02 | [-0.08, 0.12] | 0.03 | [-0.07, 0.13] |
| Xanthurenic acid | 0.03 | [-0.10, 0.16] | 0.04 | [-0.08, 0.16] |
| Quinolinic acid | **0.08 *** | **[0.01, 0.15]** | 0.07 | [-0.00, 0.14] |
| Picolinic acid | 0.03 | [-0.09, 0.14] | 0.03 | [-0.09, 0.15] |
| Nicotinic acid | 0.09 | [-0.20, 0.39] | 0.12 | [-0.17, 0.42] |
| All continuous predictors are mean-centered and scaled by 1 standard deviation. The dependent variables are in the log conversed value and N = 181 for all except for nicotinic acid N = 165. *** p < 0.001; ** p < 0.01; * p < 0.05. | | | | |

**Supplemental Table S13.** Clinical descriptives of the amniotic infection syndrome (AIS) cohort and their matched controls.

| **Variable** | **Control (n = 94)** | **AIS (n = 47)** | **P-value** |
| --- | --- | --- | --- |
| **Age mom** | 29.5 (27.0, 33.0) | 29.0 (27.0, 32.0) | 0.642 |
| **BMI (kg/m^2^)** | 23.7 (21.2, 26.8) | 23.9 (21.3, 26.8) | 0.771 |
| **Smoking** |  |  | 0.143 |
| never | 45 (49.5%) | 18 (40.9%) |  |
| former | 42 (46.2%) | 20 (45.5%) |  |
| last 4 weeks of pregnancy | 4 (4.4%) | 6 (13.6%) |  |
| **Cesarean section** | 23 (24.7%) | 15 (32.6%) | 0.327 |
| **Gestational age (weeks)** | 39.0 (37.0, 40.0) | 39.0 (36.0, 40.0) | 0.648 |
| **Sex girl** | 34 (36.2%) | 17 (36.2%) | 1 |
| **Birth weight (grams)** | 3345.0 (2847.5, 3607.5) | 3325.0 (2570.0, 3812.5) | 0.756 |
| **Placenta weight (grams)** | 550.0 (490.0, 630.0) | 520.0 (460.0, 607.5) | 0.163 |
| **NICU admission** | 20 (21.7%) | 15 (32.6%) | 0.167 |
| **Preeclampsia** | 0 (0.0%) | 4 (8.5%) |  |
| **FGR** | 0 (0.0%) | 3 (6.4%) |  |
| **GDM** | 0 (0.0%) | 4 (8.5%) |  |
| **DM** | 0 (0.0%) | 1 (2.1%) |  |
| **AIS** | 0 (0.0%) | 17 (100.0%) |  |

Abbreviations: BMI, body mass index; NICU, neonatal intensive care unit; FGR, fetal growth restriction; GDM, gestational diabetes mellitus; DM, diabetes mellitus before pregnancy; AIS, amniotic infection syndrome.

**Supplemental Table S14.** Umbilical cord blood KP metabolite concentrations in the amniotic infection syndrome (AIS) cohort and their matched controls.

| **Variable** | **Control (n = 94)** | **AIS (n = 47)** | **P value** | **Adj. P** |
| --- | --- | --- | --- | --- |
| Tryptophan | 70826 (64357, 77090) | 70548 (60950, 79789) | 0.584 | 0.933 |
| Kynurenine | 4965 (4311, 5557) | 4770 (4255, 5837) | 0.933 | 0.933 |
| Anthranilic acid | 31 (26, 42) | 36 (27, 46) | 0.666 | 0.933 |
| 3-Hydroxykynurenine | 104 (84, 126) | 113 (82, 140) | 0.834 | 0.933 |
| 3-Hydroxyanthranilic acid | 255 (166, 345) | 255 (143, 370) | 0.911 | 0.933 |
| Kynurenic acid | 369 (290, 465) | 361 (284, 473) | 0.592 | 0.933 |
| Xanthurenic acid | 35 (26, 48) | 37 (26, 52) | 0.68 | 0.933 |
| Quinolinic acid | 1599 (1318, 1922) | 1721 (1394, 2022) | 0.352 | 0.933 |
| Picolinic acid | 73 (54, 95) | 85 (60, 101) | 0.419 | 0.933 |
| Nicotinic acid | 1 (0, 3) | 1 (0, 2) | 0.711 | 0.933 |
| * Concentrations are depicted in nmol/l as median (Q1, Q3). The P values were calculated using t-tests adjusted with BH correction (adj. P) on the natural log transformed data. | | | | |

**Supplemental Table S15.** Linear regression table depicting the effect of amniotic infection syndrome (AIS) on the umbilical cord blood concentrations of KP metabolites. Model 1 is only adjusted for the preeclampsia. Model 2 is additionally adjusted for fetal sex, as well as maternal BMI, gestational age and smoking status.

|  | **Model 1** | | **Model 2** | |
| --- | --- | --- | --- | --- |
| **Dependent** | **Estimate** | **95% CI** | **Estimate** | **95% CI** |
| Tryptophan | 0.00 | [-0.06, 0.06] | -0.01 | [-0.07, 0.06] |
| Kynurenine | 0.01 | [-0.06, 0.08] | 0.02 | [-0.06, 0.10] |
| Anthranilic Acid | 0.05 | [-0.08, 0.18] | 0.08 | [-0.05, 0.22] |
| 3-Hydroxy kynurenine | 0.03 | [-0.13, 0.18] | 0.01 | [-0.15, 0.17] |
| 3-Hydroxy anthranilic acid | 0.02 | [-0.21, 0.24] | -0.02 | [-0.24, 0.19] |
| Kynurenic acid | -0.01 | [-0.14, 0.12] | 0.01 | [-0.11, 0.13] |
| Xanthurenic acid | 0.03 | [-0.14, 0.20] | 0.05 | [-0.11, 0.22] |
| Quinolinic acid | 0.07 | [-0.05, 0.18] | 0.08 | [-0.03, 0.19] |
| Picolinic acid | 0.06 | [-0.08, 0.20] | 0.10 | [-0.05, 0.25] |
| Nicotinic acid | 0.05 | [-0.47, 0.56] | 0.06 | [-0.49, 0.62] |
| All continuous predictors are mean-centered and scaled by 1 standard deviation. The dependent variables are in the log conversed value and N = 138 for all except for nicotinic acid N = 118. *** p < 0.001; ** p < 0.01; * p < 0.05. | | | | |

**Supplemental Figure S1. The associations between gestational age and kynurenine pathway (KP) metabolites in umbilical cord blood.** Correlations between gestational age and each KP metabolite were tested on the full cohort (n=615) using Pearson correlation analysis.

**
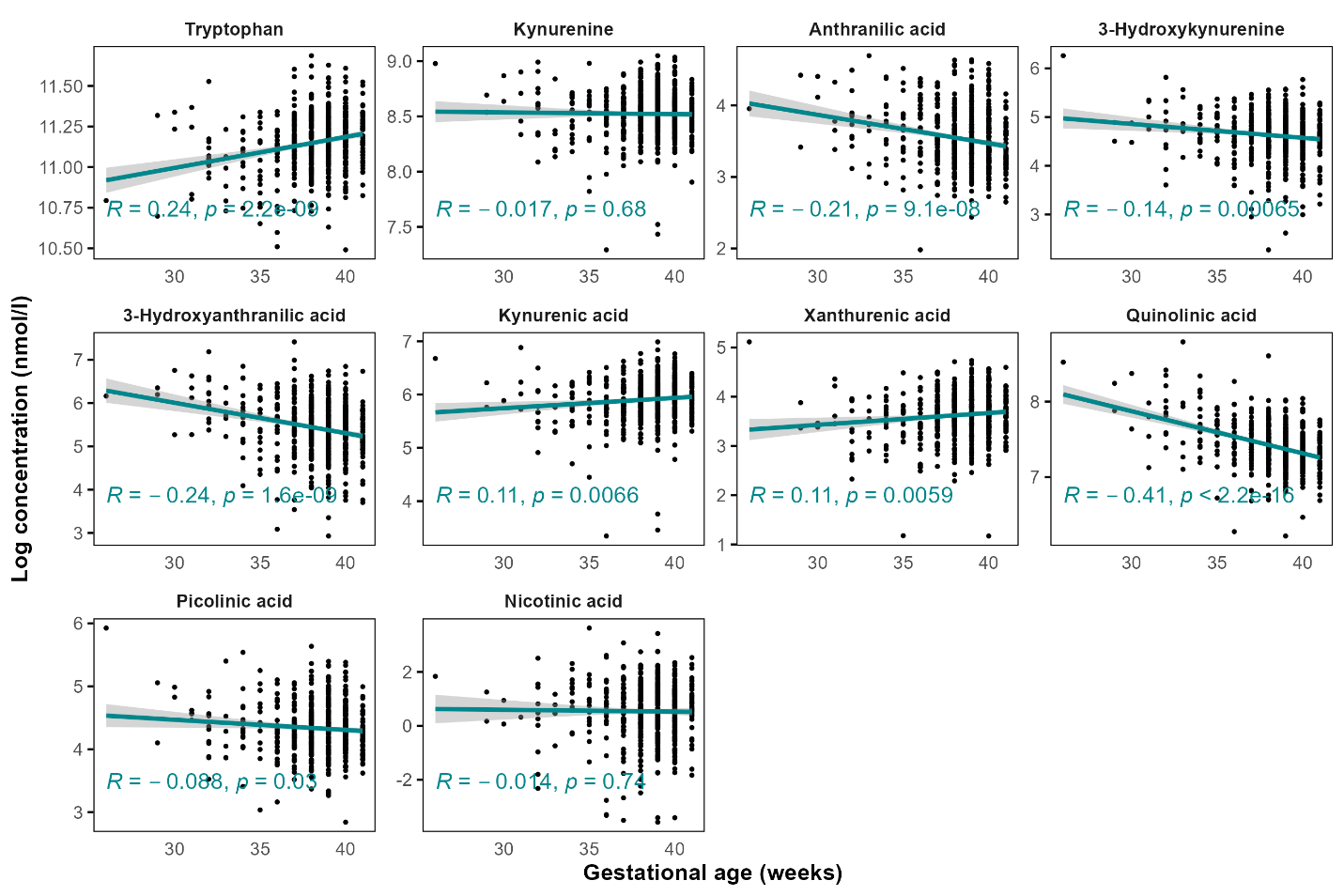
**

**Supplemental Figure S2. The associations between body mass index (BMI) and kynurenine pathway (KP) metabolites in umbilical cord blood.** Correlations between BMI and each KP metabolite were tested on the full cohort minus one univariate extreme BMI outlier (n=614) using Pearson correlation analysis.

**
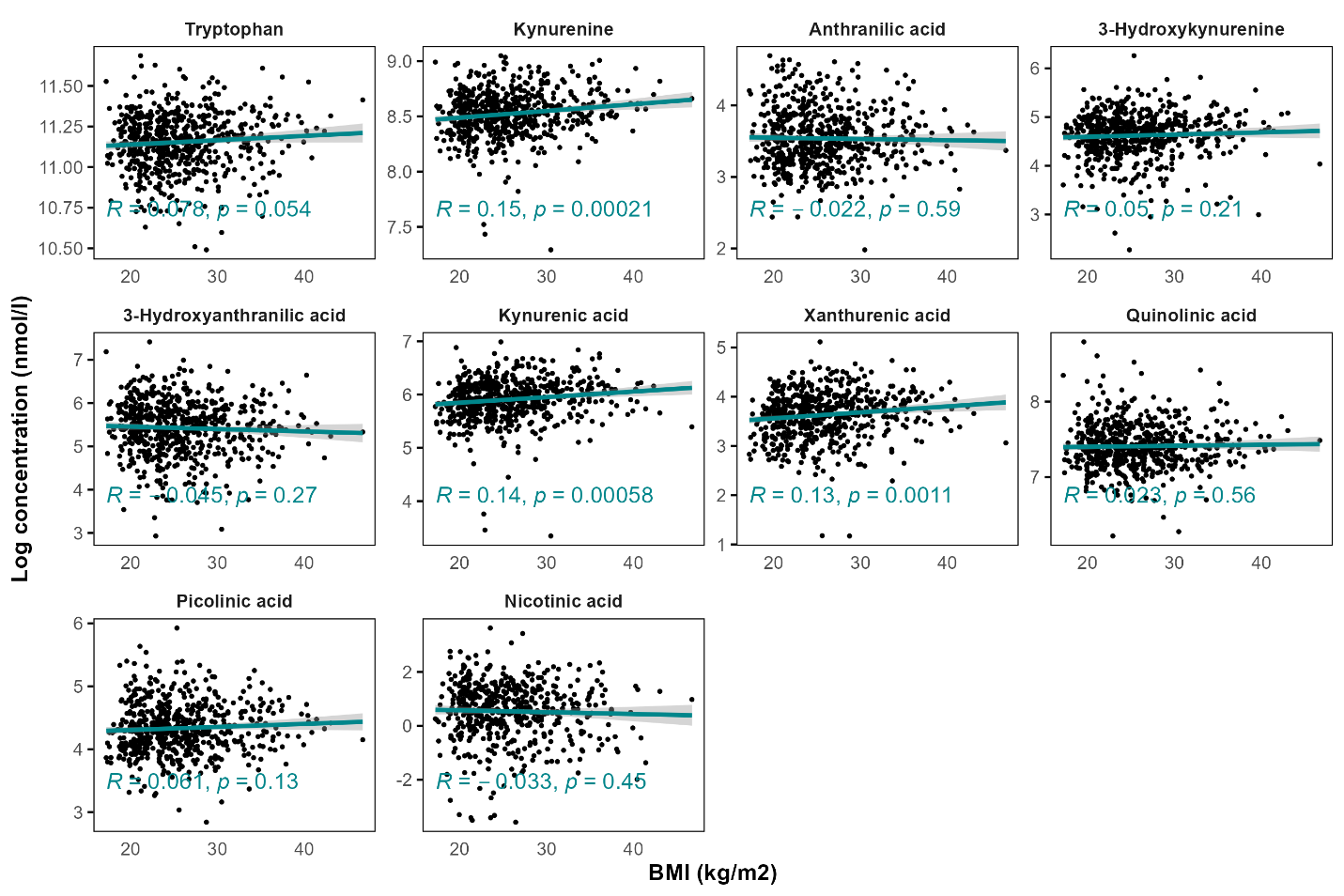
**

**Supplemental Figure S3. The associations between fetal sex and kynurenine pathway metabolites concentrations in umbilical cord blood.** Differences in concentrations between boys and girls were statistically tested for each kynurenine pathway metabolite using a t-test. N = 615, ** P < 0.01, *** P < 0.001.


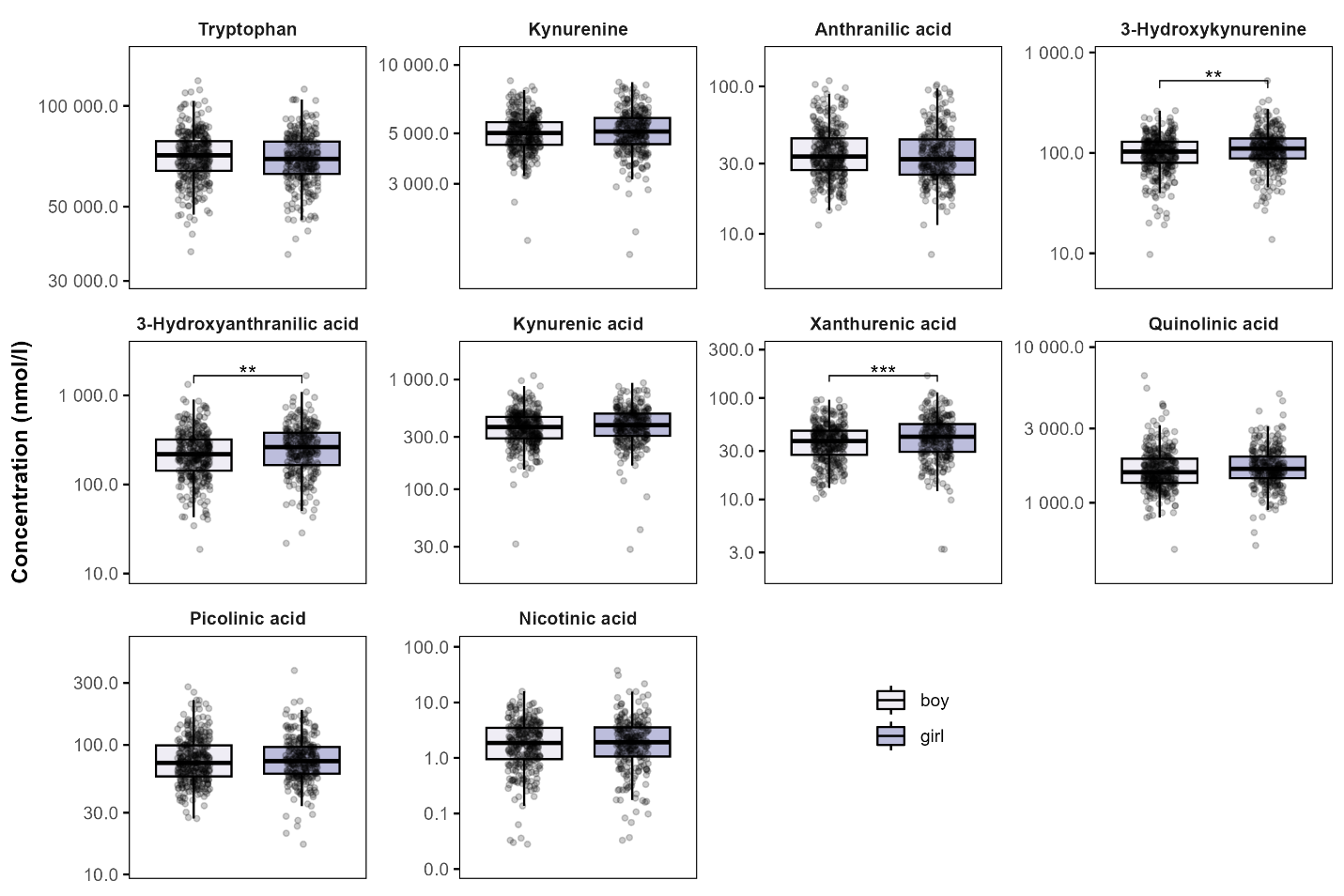


**Supplemental Figure S4. The associations between maternal smoking status and kynurenine pathway metabolites concentrations in umbilical cord blood.** Differences in concentrations between smoking status were statistically tested for each kynurenine pathway metabolite using an ANOVA followed by t-tests between groups of the statistically significant metabolites. N = 615, * P < 0.05, ** P < 0.01.


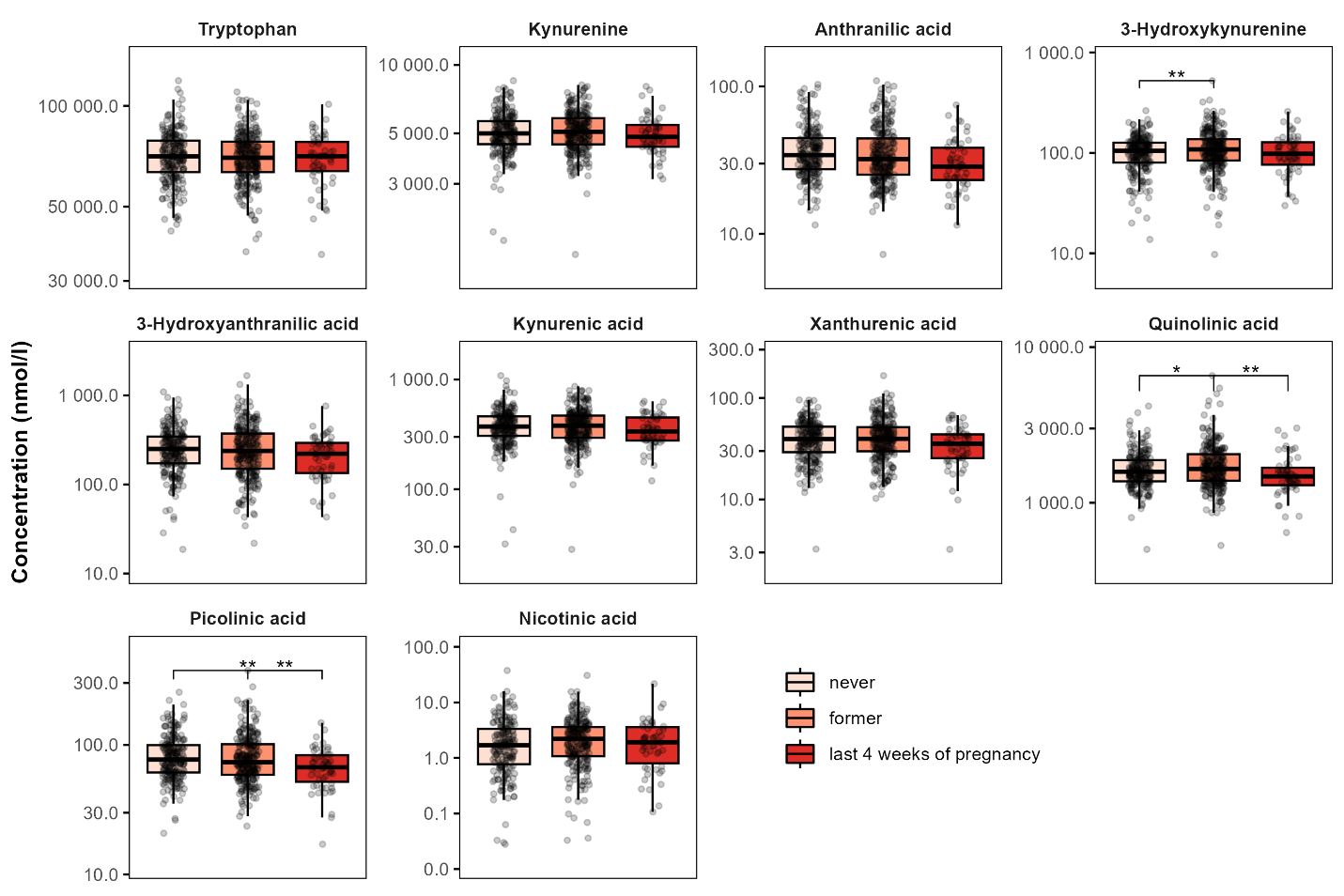


**Supplemental Figure S5. The associations between mode of delivery and kynurenine pathway metabolites concentrations in umbilical cord blood.** Differences in concentrations between caesarean section (sectio) and spontaneous delivery (spontaneous) were statistically tested for each kynurenine pathway metabolite using a t-test. ** P < 0.01, *** P < 0.001, **** P < 00001.


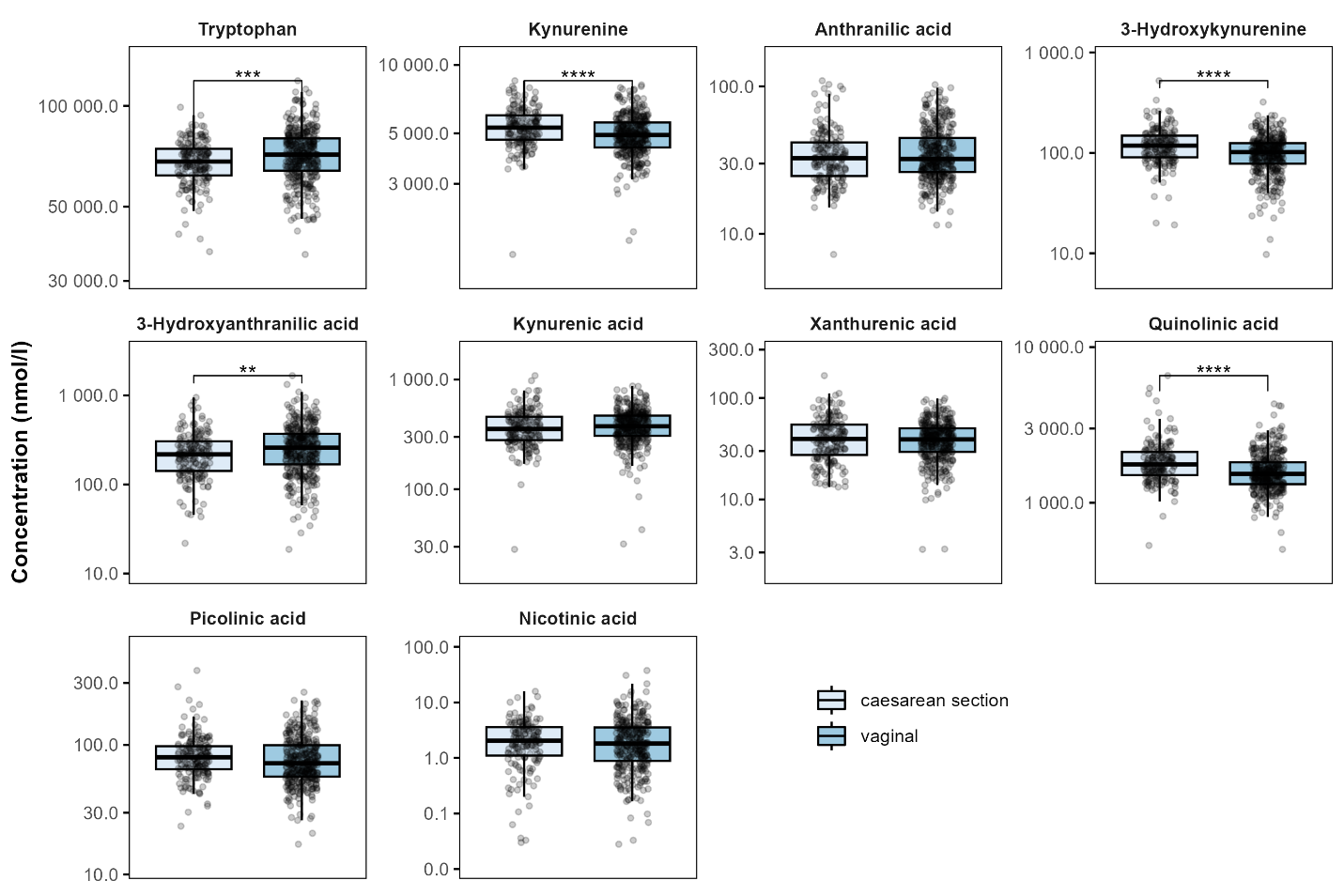


**Supplemental Figure S6. The associations between maternal age and kynurenine pathway (KP) metabolites in umbilical cord blood.** Correlations between age and each KP metabolite were tested on the full cohort minus two with missing ages (n=613) using Pearson correlation analysis.

**
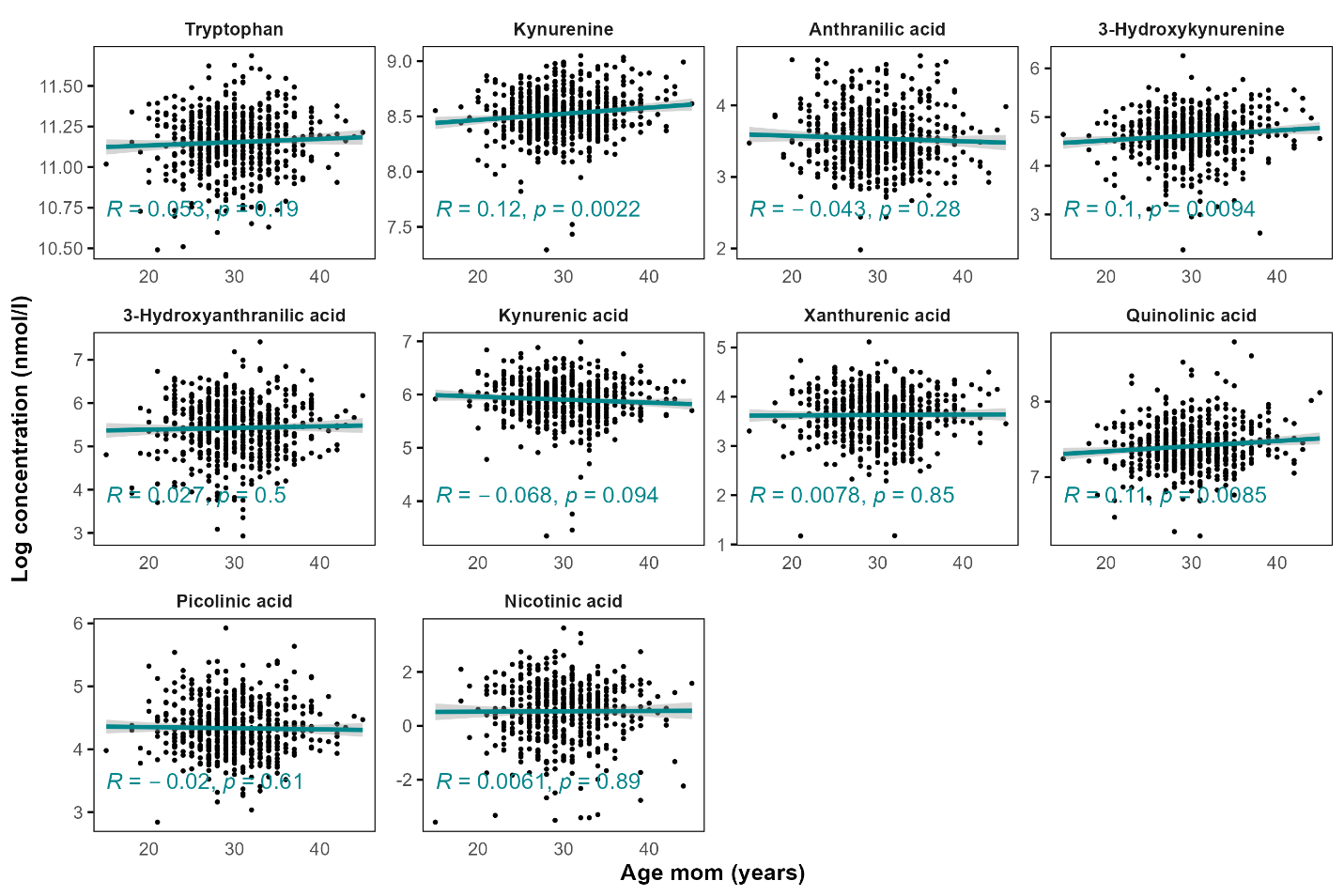
**

**Supplemental Figure S7. The effect of preeclampsia (PE) on correlations between kynurenine pathway (KP) metabolites in umbilical cord blood.** The top panels display the correlation coefficients split by controls (**A**) and preeclampsia (**B**). The crosses (X) denote absence of a statistically significant correlation.

**
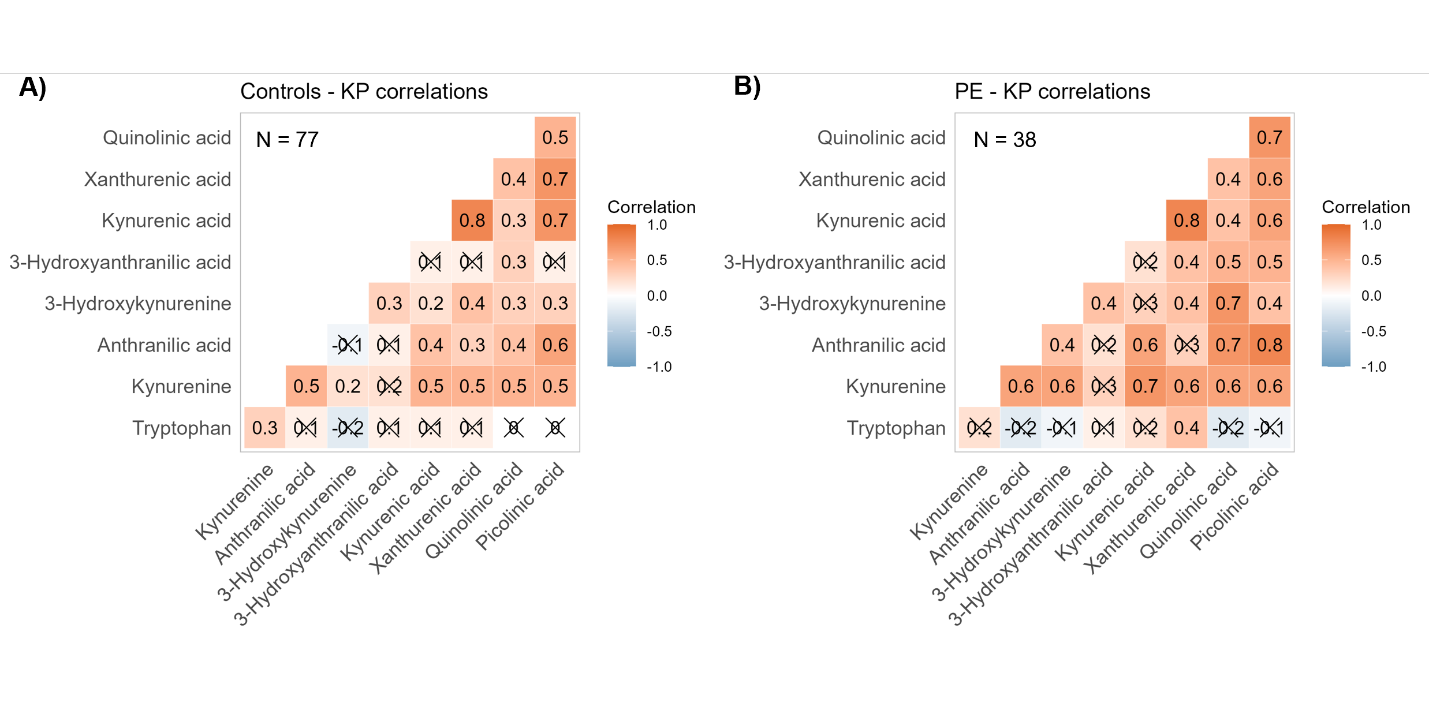
**

**Supplemental Figure S8. The effect of fetal growth restriction (FGR) on correlations between kynurenine pathway (KP) metabolites in umbilical cord blood.** The top panels display the correlation coefficients split by controls (**A**) and FGR (**B**). The crosses (X) denote absence of a statistically significant correlation.

**
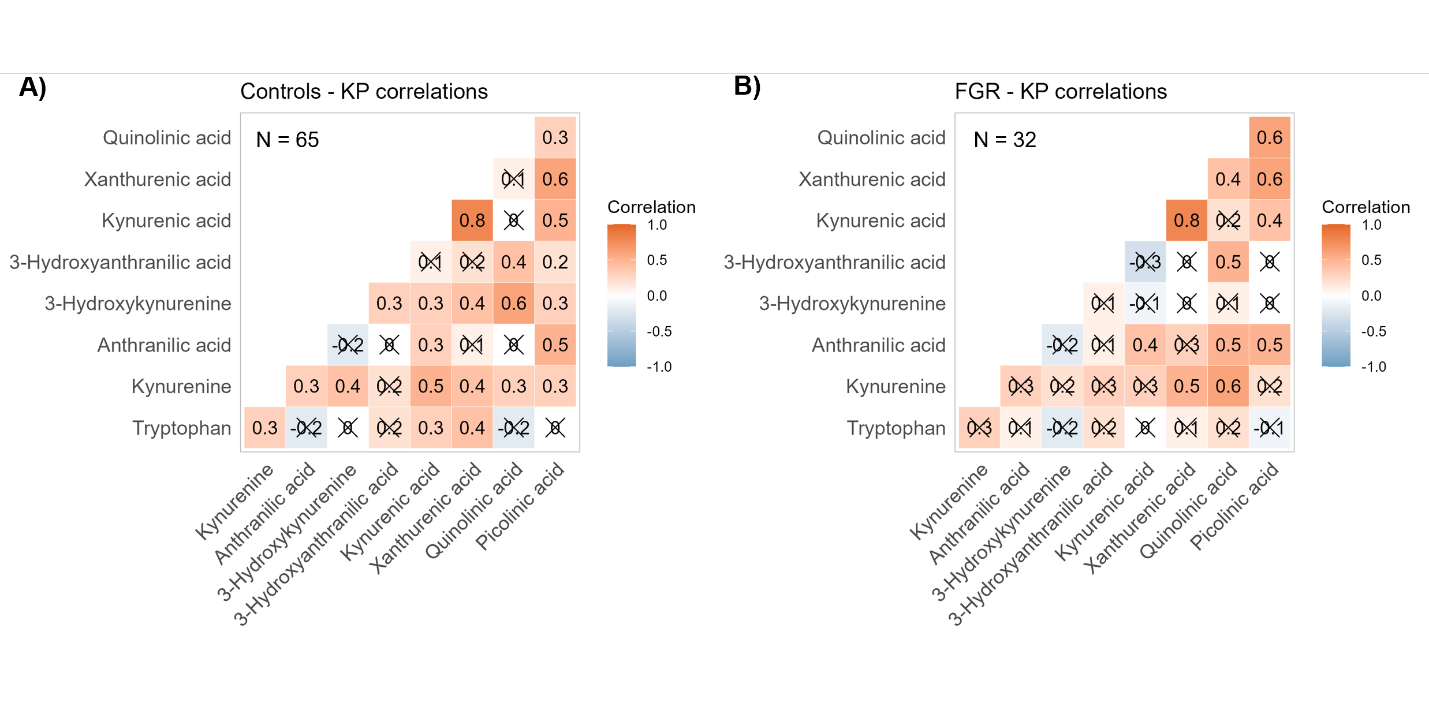
**

**Supplemental Figure S9. The effect of diabetes mellitus before pregnancy (DM) on correlations between kynurenine pathway (KP) metabolites in umbilical cord blood.** The top panels display the correlation coefficients split by controls (**A**) and DM (**B**). The crosses (X) denote absence of a statistically significant correlation.**
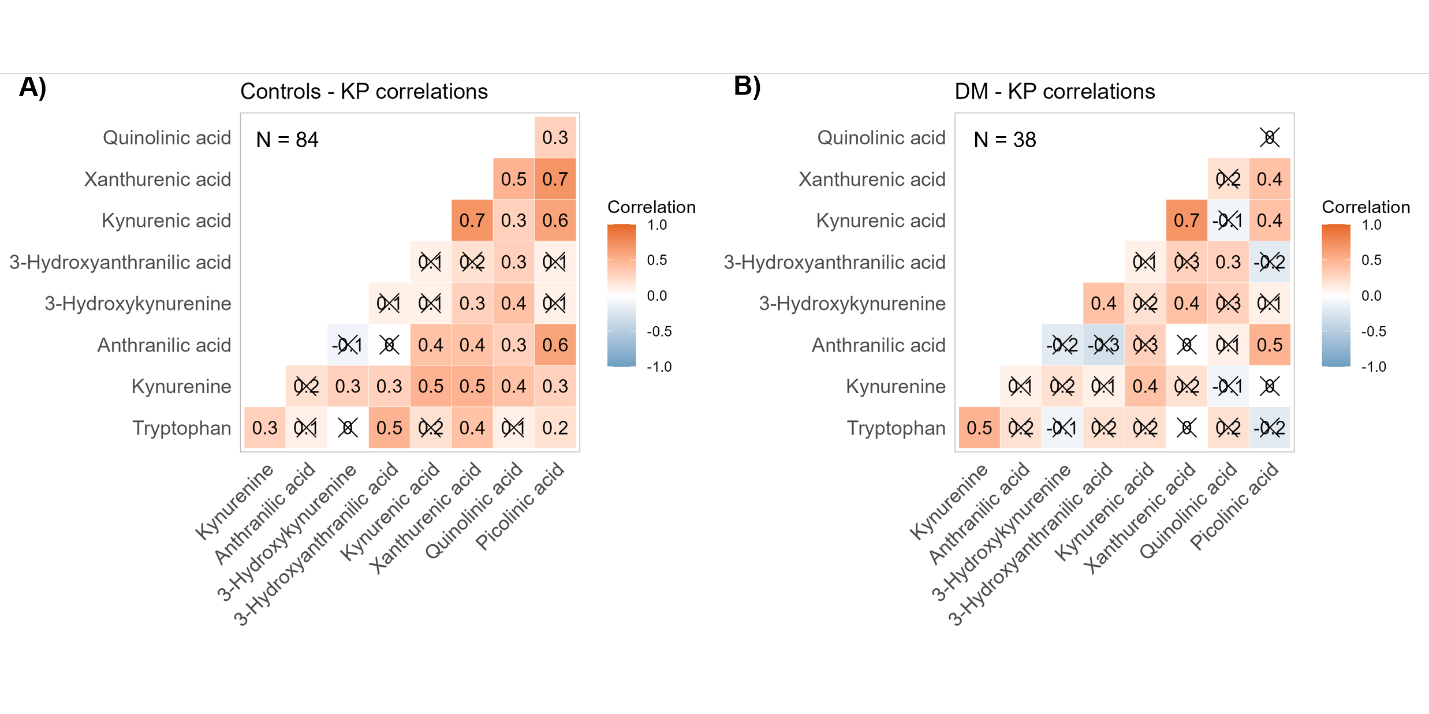
**

**Supplemental Figure S10. The effect of gestational diabetes mellitus (GDM) on correlations between kynurenine pathway (KP) metabolites in umbilical cord blood.** The top panels display the correlation coefficients split by controls (**A**) and GDM (**B**). The crosses (X) denote absence of a statistically significant correlation.

**
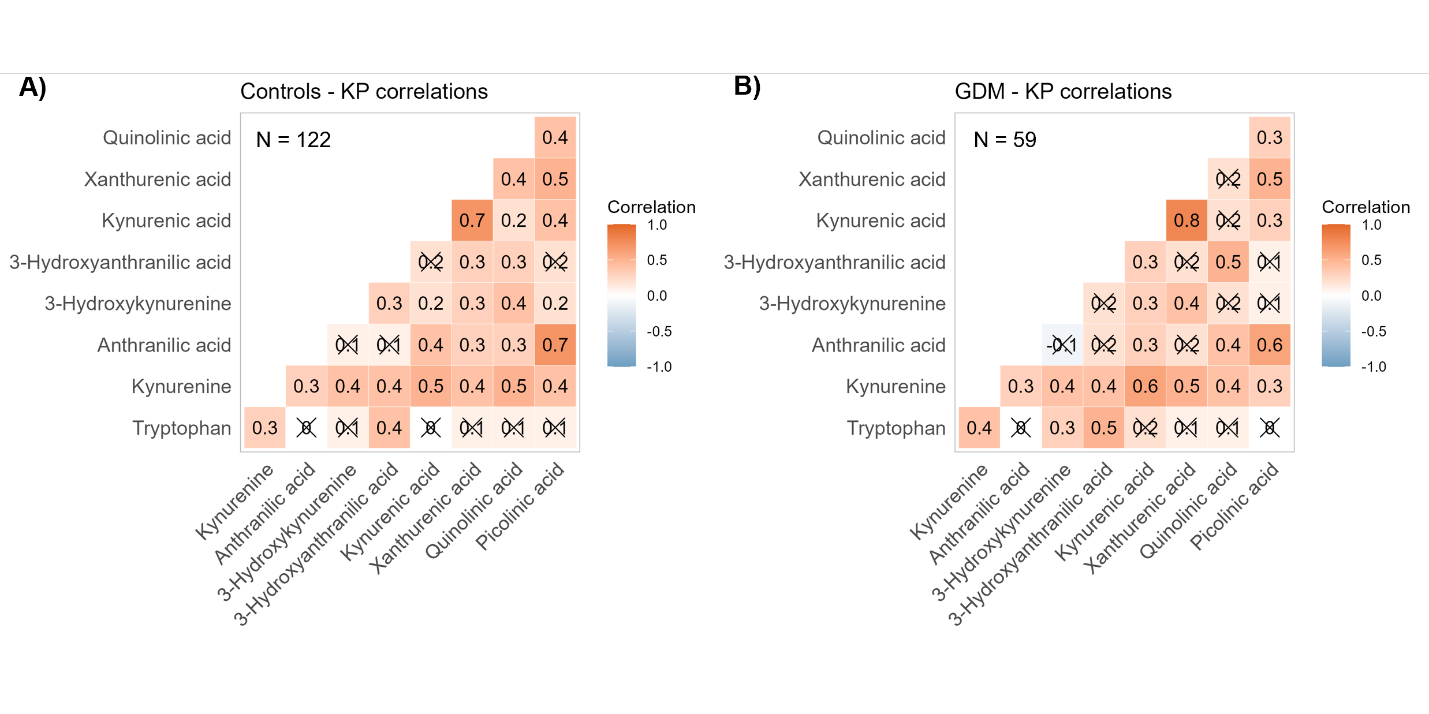
**

**Supplemental Figure S11. The effect of amniotic infection syndrome (AIS) on correlations between kynurenine pathway (KP) metabolites in umbilical cord blood.** The top panels display the correlation coefficients split by controls (**A**) and AIS (**B**). The crosses (X) denote absence of a statistically significant correlation.

**
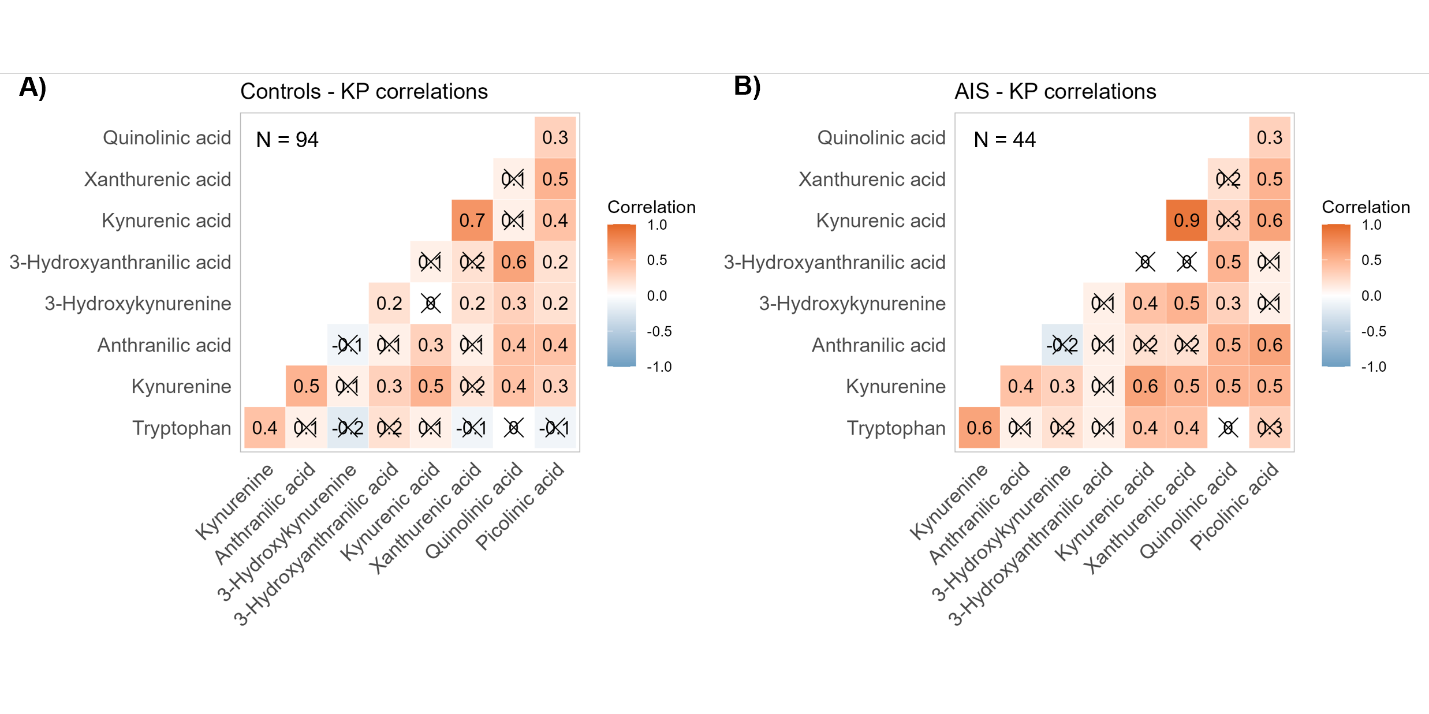
**
